# Vascular mechanisms of post-COVID-19 conditions: Rho-kinase is a novel target for therapy

**DOI:** 10.1093/ehjcvp/pvad025

**Published:** 2023-04-05

**Authors:** Robert A Sykes, Karla B Neves, Rhéure Alves-Lopes, Ilaria Caputo, Kirsty Fallon, Nigel B Jamieson, Anna Kamdar, Assya Legrini, Holly Leslie, Alasdair McIntosh, Alex McConnachie, Andrew Morrow, Richard W McFarlane, Kenneth Mangion, John McAbney, Augusto C Montezano, Rhian M Touyz, Colin Wood, Colin Berry

**Affiliations:** School of Cardiovascular and Metabolic Health, University of Glasgow, UK; West of Scotland Heart and Lung Centre, Golden Jubilee National Hospital, Glasgow, UK; School of Cardiovascular and Metabolic Health, University of Glasgow, UK; Institute of Pharmacy and Biomedical Sciences, University of Strathclyde, Glasgow, UK; School of Cardiovascular and Metabolic Health, University of Glasgow, UK; Università degli Studi di Padova, 35122 Padova, Italy; Clinical Research Facility, Queen Elizabeth University Hospital, NHS Greater Glasgow & Clyde Health Board, Glasgow, UK; Wolfson Wohl Cancer Research Centre, School of Cancer Sciences, University of Glasgow, Glasgow, UK; School of Cardiovascular and Metabolic Health, University of Glasgow, UK; Wolfson Wohl Cancer Research Centre, School of Cancer Sciences, University of Glasgow, Glasgow, UK; Wolfson Wohl Cancer Research Centre, School of Cancer Sciences, University of Glasgow, Glasgow, UK; Robertson Centre for Biostatistics, School of Health and Wellbeing, University of Glasgow, Glasgow, UK; Robertson Centre for Biostatistics, School of Health and Wellbeing, University of Glasgow, Glasgow, UK; School of Cardiovascular and Metabolic Health, University of Glasgow, UK; West of Scotland Heart and Lung Centre, Golden Jubilee National Hospital, Glasgow, UK; School of Cardiovascular and Metabolic Health, University of Glasgow, UK; School of Cardiovascular and Metabolic Health, University of Glasgow, UK; Department of Cardiology, Queen Elizabeth University Hospital, NHS Greater Glasgow and Clyde Health Board, Glasgow, UK; Institute of Biomedical and Life Sciences (FBLS), University of Glasgow, Glasgow G12 8QQ, UK; School of Cardiovascular and Metabolic Health, University of Glasgow, UK; Research Institute of the McGill University Health Centre (RI-MUHC), Montreal, QC H4A 3J1, Canada; School of Cardiovascular and Metabolic Health, University of Glasgow, UK; Research Institute of the McGill University Health Centre (RI-MUHC), Montreal, QC H4A 3J1, Canada; Wolfson Wohl Cancer Research Centre, School of Cancer Sciences, University of Glasgow, Glasgow, UK; School of Cardiovascular and Metabolic Health, University of Glasgow, UK; West of Scotland Heart and Lung Centre, Golden Jubilee National Hospital, Glasgow, UK; Department of Cardiology, Queen Elizabeth University Hospital, NHS Greater Glasgow and Clyde Health Board, Glasgow, UK

**Keywords:** Calcium signalling, Smooth muscle cells, COVID-19, Vascular dysfunction, Endothelium-independent dysfunction, Rho-kinase

## Abstract

**Background:**

In post-coronavirus disease-19 (post-COVID-19) conditions (long COVID), systemic vascular dysfunction is implicated, but the mechanisms are uncertain, and the treatment is imprecise.

**Methods and results:**

Patients convalescing after hospitalization for COVID-19 and risk factor matched controls underwent multisystem phenotyping using blood biomarkers, cardiorenal and pulmonary imaging, and gluteal subcutaneous biopsy (NCT04403607). Small resistance arteries were isolated and examined using wire myography, histopathology, immunohistochemistry, and spatial transcriptomics. Endothelium-independent (sodium nitroprusside) and -dependent (acetylcholine) vasorelaxation and vasoconstriction to the thromboxane A2 receptor agonist, U46619, and endothelin-1 (ET-1) in the presence or absence of a RhoA/Rho-kinase inhibitor (fasudil), were investigated. Thirty-seven patients, including 27 (mean age 57 years, 48% women, 41% cardiovascular disease) 3 months post-COVID-19 and 10 controls (mean age 57 years, 20% women, 30% cardiovascular disease), were included. Compared with control responses, U46619-induced constriction was increased (*P* = 0.002) and endothelium-independent vasorelaxation was reduced in arteries from COVID-19 patients (*P* < 0.001). This difference was abolished by fasudil. Histopathology revealed greater collagen abundance in COVID-19 arteries {Masson's trichrome (MT) 69.7% [95% confidence interval (CI): 67.8–71.7]; picrosirius red 68.6% [95% CI: 64.4*–*72.8]} vs. controls [MT 64.9% (95% CI: 59.4–70.3) (*P* = 0.028); picrosirius red 60.1% (95% CI: 55.4–64.8), (*P* = 0.029)]. Greater phosphorylated myosin light chain antibody-positive staining in vascular smooth muscle cells was observed in COVID-19 arteries (40.1%; 95% CI: 30.9–49.3) vs. controls (10.0%; 95% CI: 4.4–15.6) (*P* < 0.001). In proof-of-concept studies, gene pathways associated with extracellular matrix alteration, proteoglycan synthesis, and viral mRNA replication appeared to be upregulated.

**Conclusion:**

Patients with post-COVID-19 conditions have enhanced vascular fibrosis and myosin light change phosphorylation. Rho-kinase activation represents a novel therapeutic target for clinical trials.

## Graphical Abstract

**Graphical Abstract:**
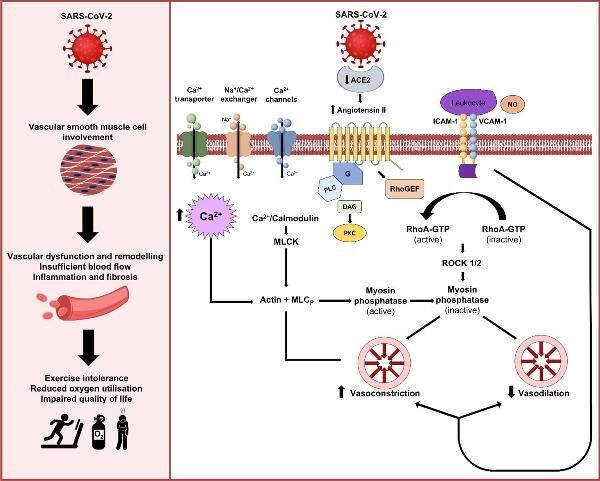
Schematic illustration of the potential mechanisms affecting vascular smooth muscle cells and systemic vascular dysfunction that may contribute to impaired functional status and aerobic exercise capacity in post-COVID-19 conditions.

## Background

The global burden of persisting illness after coronavirus disease-19 (COVID-19) is estimated to include 144.7 million patients, representing 3.7% of all infections.^1^ The illness trajectory of post-COVID-19 conditions (long COVID) differs between community and hospitalized populations,^2,3,4^ and persisting symptoms are more common in hospitalized patients (52% vs. 38%).^5^ Cardiovascular symptoms include dyspnoea, lethargy, and chest pain, leading to exercise limitation; residual lung disease may not account for these symptoms.^4^

Cardiovascular involvement during acute COVID-19 occurs in approximately one in eight hospitalized patients.^4^ Vascular involvement may include endotheliitis, thrombo-embolic microvascular burden, inflammation, and oxidative stress.^6^ In addition, deconditioning and muscle wasting may further exacerbate symptoms and prolong recuperation.^7^ Severe acute respiratory syndrome coronavirus 2 (SARS-CoV-2) infects cells by binding to angiotensin-converting enzyme-2 (ACE2) on the cell membrane.^8^ ACE2 is widely distributed in systemic tissues, including the lung and cardiovascular system.^9^ SARS-CoV-2 binding reduces ACE2 expression and impairs ACE2 function leading to endothelial dysfunction,^10,11^ manifesting through impaired nitric oxide (NO) production and haemostasis activation.^12,13^

Small resistance arteries are the final common pathway for delivering oxygenated blood and nutrients to tissues. Vascular tone is regulated by endothelial-mediated vasorelaxation and vascular smooth muscle cell (VSMC)-mediated constriction. RhoA/Rho-kinase signalling pathways control VSMC contraction, migration, and growth, and increased Rho-kinase activity is evident in models of vascular dysfunction.^14,15^ The potential antiviral effects of Rho-kinase inhibition as a treatment for acute COVID-19 have been postulated^16–19^; however, there are no reported data on Rho-kinase inhibition for the treatment of post-COVID-19 conditions. Endothelial dysfunction^10,11^ is implicated in post-COVID-19 conditions. However, the role of non-endothelial pathways and VSMCs is unknown.

The main aim of our research is to identify vascular mechanisms that may represent druggable targets for therapy development in post-COVID-19 conditions. We hypothesized that SARS-CoV-2 infection impairs non-endothelium-dependent vasorelaxation pathways through VSMC and RhoA/Rho-kinase activation, which, together with altered calcium ion (Ca^2+^) handling in these cells, impairs vascular function in patients with post-COVID-19 conditions, compared with matched controls.

To investigate this hypothesis, we pre-defined a mechanistic investigation within a prospective clinical study.

## Methods

### Study approval

Ethical approval for the CISCO-19 study and gluteal biopsy sub-study was obtained from the UK National Research Ethics Service (Reference 20/NS/0066). Informed written consent and continued eligibility assessment were obtained before conducting study procedures.

### Patient recruitment and clinical features

We undertook a prospective, observational, multicentre, secondary care cohort study assessing the prevalence and clinical significance of multi-organ injury in survivors of COVID-19 during convalescence.^4,20^ Participating patients were invited to undergo a gluteal biopsy to obtain small arteries for *in vitro* studies *ex vivo*. The methodology, including biomarkers, patient-reported outcome measures, cardiovascular computed tomography angiography, and cardio-renal magnetic resonance imaging, is described within the supplement.^20^

Control patients who had received secondary care and had similar age, sex, and cardiovascular morbidities were prospectively screened and invited to participate. They were confirmed to be COVID-19 antibody-negative using the Roche^®^ Elecsys anti-SARS-CoV-2 S quantitative assay without previous positive polymerase chain reaction (PCR) positivity or history consistent with COVID-19.

### Gluteal biopsies

Patient volunteers were made comfortable lying prone. Local anaesthetic (2% lidocaine) was carefully administered in a sterile surgical field in the gluteal area. A 4–6 cm^2^ sample of subcutaneous fat with 0.4 cm^2^ skin was excised and submerged in a physiological saline solution. Intact small arteries (<500 μm) were dissected from this subcutaneous fat. These arteries were used for histopathology, functional (wire myography), and molecular studies, and VSMCs were isolated for primary cell culture, as previously described and summarized below.^21^ Identical protocols were used for laboratory studies in tissues obtained from post-COVID-19 patients and controls. Pharmacological assessment of peripheral vascular function was performed at least 3 months after hospitalization for COVID-19.

### Human vascular functional studies

Small arteries were dissected from gluteal fat and cut into 2-mm ring segments. Arterial segments were mounted on isometric wire myographs (Danish Myo Technology, Denmark) as described within the supplement. Following 30 min of equilibration, the contractile responses of arterial segments were assessed by adding KCl (62.5 mmol/L). Arteries with no responses were retained for cell culture or molecular studies. The integrity of the endothelium was verified by relaxation induced by acetylcholine (ACh, 10^−6^ mol/L) in arteries pre-contracted with U46619 (thromboxane-A2 analogue, 10^−7^ mol/L). Cumulative concentration–response curves (CCRCs) were constructed for endothelium-dependent relaxation to ACh (10^−9^–3 × 10^−5^ mol/L). Concentration–response curves assessed endothelium-independent vasorelaxation to sodium nitroprusside (SNP; 10^−10^–10^−5^ mol/L) in human vessels. Concentration–response curves to U46619 (10^−10^–10^−6^ mol/L) and endothelin-1 (ET-1; 10^−12^–10^−7^ mol/L) were performed to evaluate vasoconstriction in human arteries. Vascular functional responses were also assessed in the absence and presence of a Rho-kinase inhibitor, fasudil (Asahi Kasei Corporation) (10^−6^ mol/L, 30 min). The vascular sensitivity (pEC_50_) and maximum responses (*E*_max_) to each agonist were determined using LabChart^®^ ADInstruments.

### Histopathology and immunohistochemistry

Fresh, vascular samples were formalin-fixed and impregnated with paraffin before staining. Analyses were undertaken using a de-identified dataset blind to COVID-19 status and the results of the other vascular investigations performed.

#### Histopathology

Masson's trichrome staining was used to selectively stain connective tissue, including collagen, from cells. Wiegert's haematoxylin was used to stain cell nuclei. Plasma stain was then applied, followed by phosphomolybdic acid and aniline blue. Picrosirius red staining of collagen I and III fibres was also performed, with celestine blue staining, Wiegert's haematoxylin, acid alcohol differentiation, and Sirius Red stain. Slides were scanned in high resolution for digital analysis in ImageJ (Fiji v1.53f51) at ×40 magnification. Colour deconvolution for Masson’s trichrome and picrosirius red was performed with threshold adjustment to assess the percentage of stain by colour for each vascular sample (Supplementary material online, *Figure S1*). The proportion of aniline blue or picrosirius red stained tissue from all tissue within the region of interest was then calculated and compared between post-COVID-19 and control samples.

#### Antigen retrieval and immunohistochemistry

Heat-induced epitope retrieval was performed for antigen retrieval, with sections treated at full pressure with the Access Retrieval Unit (Menarini) in a sodium citrate buffer for anti-myosin light chain (phosphor S20) antibody (Abcam 2480). Sections were then washed in Tris Tween buffer.

Hydrogen peroxide (3%) treatment was then applied in phosphate-buffered saline to quench peroxidase activity, followed by two further washes with TRIS Tween buffer. Sections were then incubated at room temperature for 30 min with the primary antibody anti-myosin chain (Abcam) at a 1:400 concentration. A further wash with TRIS Tween buffer was then performed.

To detect primary antibodies, the sections were then incubated with EnVision+System HRP Labelled Polymer Anti-Rabbit Secondary Antibody (Dako) for 30 min at room temperature. A further washing with TRIS Tween buffer was performed followed by two 5-min incubations with 3,3′-diaminobenzidine (DAB) substrate-chromogen (EnVision+System, Dako). Sections were then rinsed twice for 5 min in distilled water prior to being counterstained using Gill's haematoxylin and mounted using DPX mounting media (Cellpath). Slides were scanned in high resolution for digital analysis in ImageJ (Fiji v1.53f51) at ×40 magnification. A colour deconvolution to calculate the total area and proportion of tissue within the slide was performed. The slide image was then reset and converted to 8-bit for threshold analysis of positively stained tissue area. The proportion of positively stained tissue to total tissue was then calculated and compared between COVID-19 and control group.

### Exploratory case-control experiments

#### Spatial transcriptomics

Spatial transcriptomics [Nanostring GeoMx Digital Spatial Profiler (DSP)] was used to assess the distribution of gene expression in small artery sections. Whole transcriptome profiling of the vascular wall was performed using formalin-fixed paraffin-embedded tissue sections (5 μm) in triplicate for two patients, one post-COVID-19 and the other being an age, sex, and cardiovascular risk factor matched control. Whole regions of interest were collected; no segmentation was performed. Bioinformatics analysis was performed on the native GeoMx DSP Data Analysis Suite before using additional custom R pipeline analysis to aid visualization. Further details are provided in the supplement.

### Statistical analysis

Cumulative concentration–response curves were fitted using a four-parameter, non-linear regression curve fitting in GraphPad Prism 8.0 (GraphPad Inc., USA). Maximum efficacy (*E*_max_) for vasoconstrictors was expressed as a percentage of the mean response of the contraction to 62.5 mM KCl. For relaxation data, the maximum response (*E*_max_) to ACh and SNP was expressed as percentage relaxation after pre-constriction with U46619 (0.1 μM). The sensitivity of the arteries to each compound was expressed as the pEC50 (constrictors) or pIC50 (inhibitors) derived from the CCRC using GraphPad Prism 8.0. The pEC50 value represents the minus log concentration required to produce 50% of the maximum response.

Similarly, the pIC50 value represents the −log of concentration required to inhibit 50% of the maximum response. Higher numbers indicate more potency (less concentration is needed to achieve the median response). The pEC50 values were calculated by computer interpolation from individual CCRCs. Statistical comparisons of continuous parameters between groups were performed using one-way and two-way ANOVA, followed by Bonferroni post-hoc tests as appropriate. Fisher's exact tests compare categorical variables within demographics and clinical data. Repeated measures ANOVA was used to compare groups within vascular reactivity studies. Mean histopathology and immunohistochemistry stain proportions were compared using an independent Student's *t*-test with 95% confidence intervals. Two-tailed significance testing with *P* < 0.05 was considered statistically significant.

### Sample size calculation

The primary endpoint of this study was the difference in maximum contraction (*E*_max_) induced by U46619 between the two groups. Using preliminary vascular reactivity data from gluteal biopsies from microvascular angina vs. control patients,^22^ we assumed a meaningful difference between the mean values in the experimental and control groups as 21.1 and standard deviation of 15. Using a significance level of 0.05 and a level of power as 80%, a minimum sample size of eight per group was estimated using G*Power 3.1 (University of Melbourne, Parkville, Victoria, Australia). This calculation was based on the Mann–Whitney U test reflecting a small sample size and the likelihood of non-parametric distribution.

## Results

### Clinical characteristics

A total of 37 patients, including 27 (mean age 57 years, 48% women, 41% cardiovascular disease) with persisting cardiovascular symptoms 3 months after hospitalization for COVID-19 and 10 controls (mean age 57 years, 20% women, 30% cardiovascular disease), were prospectively included (*Table 1*). The control patients had received hospital-based care either as inpatients or outpatients.

**Table 1:** Clinical characteristics of the population

	All	Control	COVID-19	*P*-value
	*N* = 37	*N* = 10	*N* = 27	
*Demographics*				
Age, years	57.3 ± 9.5	57.9 ± 7.8	57.1 ± 10.2	0.827
*Sex*				
Male	22 (59%)	8 (80%)	14 (52%)	0.153
Female	15 (41%)	2 (20%)	13 (48%)	
Healthcare worker	5 (14%)	1 (10%)	4 (15%)	1.000
Most deprived SIMD quintile	13 (37%)	2 (20%)	11 (44%)	0.259
*Ethnicity*				
White	35 (95%)	10 (100%)	25 (93%)	1.000
Asian	1 (3%)	0 (0%)	1 (4%)	
Other	1 (3%)	0 (0%)	1 (4%)	
*Presenting characteristics at enrolment* ^a^				
Weight, kg	92 ± 18	93 ± 12	91 ± 20	0.749
Height, cm	172 ± 9	176 ± 8	170 ± 9	0.086
Body mass index, kg/m^2^	31.1 ± 5.8	30.2 ± 4.1	31.4 ± 6.4	0.567
Body surface area, m^2^	2.1 ± 0.2	2.1 ± 0.2	2.1 ± 0.3	0.447
Heart rate, b.p.m.	88 ± 21	67 ± 8	97 ± 19	<0.001
Systolic blood pressure, mmHg	132 ± 17	137 ± 18	130 ± 17	0.266
Diastolic blood pressure, mmHg	76 ± 12	80 ± 13	75 ± 11	0.204
Peripheral oxygen saturation, %	94 ± 8	98 ± 1	92 ± 9	0.055
Respiratory rate, per min	20 ± 7	13 ± 2	22 ± 6	<0.001
*WHO clinical severity score*				
Hospitalized, no oxygen therapy	7 (26%)	—	7 (26%)	—
Oxygen by mask or nasal prongs	12 (44%)	—	12 (44%)	
Non-invasive ventilation	2 (7%)	—	2 (7%)	
Mechanical ventilation	6 (22%)	—	6 (22%)	
*COVID-19 diagnosis*				
PCR test	27 (73%)	0 (0%)	27 (100%)	—
Nosocomial	1 (3%)	0 (0%)	1 (4%)	—
*Radiology, chest radiograph, or CT scan*				
Typical of COVID-19	20 (80%)	—	20 (80%)	—
Atypical of COVID-19	0 (0%)	—	0 (0%)	
Unlikely	0 (0%)	—	0 (0%)	
Normal	5 (20%)	—	5 (20%)	
*Acute COVID-19 therapy*				
Oxygen	20 (74%)	—	20 (74%)	—
Steroid	15 (56%)	—	15 (56%)	—
Antiviral	9 (33%)	—	9 (33%)	—
Non-invasive respiratory support	6 (22%)	—	6 (22%)	—
Intensive care	9 (33%)	—	9 (33%)	—
Invasive ventilation	5 (19%)	—	5 (19%)	—
Intravenous inotrope	3 (11%)	—	3 (11%)	—
*Cardiovascular history*				
*Smoking*				
Never	20 (54%)	6 (60%)	14 (52%)	0.866
Former	14 (38%)	3 (30%)	11 (41%)	
Current	3 (8%)	1 (10%)	2 (7%)	
Hypercholesterolaemia	24 (65%)	5 (50%)	19 (70%)	0.275
Hypertension	8 (22%)	2 (20%)	6 (22%)	1.000
Diabetes mellitus	8 (22%)	1 (10%)	7 (26%)	0.404
Chronic kidney disease	0 (0%)	0 (0%)	0 (0%)	1.000
*CCS angina class*				
Angina	36 (97)	10 (100%)	26 (96%)	1.000
Myocardial infarction	2 (5%)	0 (0%)	2 (7%)	1.000
Stroke or TIA	2 (5%)	0 (0%)	2 (7%)	1.000
				
Peripheral vascular disease	0 (0%)	0 (0%)	0 (0%)	1.000
Previous PCI	2 (5%)	0 (0%)	2 (7%)	1.000
Cardiovascular disease and/or treatment	14 (38%)	3 (30%)	11 (41%)	0.710
*Risk scores*				
ISARIC-4c in-hospital mortality risk, in %	10.7 ± 7.5	5.4 ± 5.4	12.7 ± 7.2	0.006
Q-Risk 3, 10-year cardiovascular risk, in %	14.5 ± 9.6	14.3 ± 9.3	14.5 ± 9.9	0.961
Charlson co-morbidity index	1.8 ± 1.4	1.3 ± 1.1	2.0 ± 1.5	0.196
*Pre-existing maintenance medication*				
Aspirin	3 (8%)	0 (0%)	3 (11%)	0.548
Statin	11 (30%)	3 (30%)	8 (30%)	1.000
Beta-blocker	4 (11%)	1 (10%)	3 (11%)	1.000
Angiotensin converting enzyme inhibitor	6 (16%)	0 (0%)	6 (22%)	0.162
Angiotensin receptor blocker	1 (3%)	0 (0%)	1 (4%)	1.000
Oral anticoagulation	0 (0%)	0 (0%)	0 (0%)	1.000
*Laboratory results, index admission*				
Initial haemoglobin, g/L	144 ± 12	143 ± 13	145 ± 13	0.772
Initial platelet count, 10^9^/L	236 ± 77	250 ± 60	231 ± 83	0.516
Initial white cell count, 10^9^/L	7.53 ± 3.80	6.48 ± 1.83	7.91 ± 4.27	0.314
Initial lymphocyte count, 10^9^/L	1.29 ± 0.64	1.91 ± 0.43	1.07 ± 0.55	0.001
Peak D-dimer, ng/mL	3931 ± 9732	195 ± 78	5052 ± 10 904	0.293
Minimum eGFR, mL/min/1.73 m^2^	76.5 ± 30.9	107.0 ± —	75.4 ± 30.9	0.325
Acute kidney injury	5 (20%)	—	5 (20%)	—
Peak hs-troponin I, ng/L	4.0 (4.0, 29.5)	4.0 (4.0, 4.0)	5.0 (4.0, 57.0)	0.187
Peak ferritin, μg/L	213 (147, 1040)	152 (97, 188)	327 (200, 1505)	0.018
Peak C-reactive protein, mg/L	72 (11, 170)	2 (1, 5)	110 (58, 186)	<0.001
Peak HbA1c, mmol/mol	46.1 ± 18.9	49.5 ± 35.5	45.1 ± 11.1	0.572
Initial albumin, g/L	35.2 ± 5.7	40.5 ± 4.0	33.2 ± 5.0	0.002
*Timelines*				
Hospitalized	25 (93%)	—	25 (93%)	—
Duration of admission, days	10 (4, 20)	— (—, —)	10 (4, 20)	—
Symptom onset to the primary outcome, days	69 (64, 74)	— (—, —)	69 (64, 74)	—
Diagnosis to the primary outcome, days	67 (62, 72)	— (—, —)	67 (62, 72)	—

Summaries are mean ± SD, median (IQR), or *N* (%). *P*-values from *t*-test, Wilcoxon–Mann–Whitney test, or Fisher's exact test. Abbreviations: SIMD, Scottish Index of Multiple Deprivation; PCR, polymerase chain reaction; CCS, Canadian Cardiovascular Society; TIA, transient ischaemic attack; PCI, percutaneous coronary intervention; CABG, coronary artery bypass graft; HbA1c, glycated haemoglobin test.

^a^Enrolment—during acute COVID-19 admission for COVID-19 group, and at attendance for screening to participate as a non-COVID-19 control for the control group.

### Multisystem phenotyping post-discharge

The clinical phenotyping was standardized to occur 28–60 days following discharge from the hospital. The median [interquartile range (IQR)] time from the date of the initial SARS-CoV-2 positive PCR result to this clinical research visit was 68 (61, 77) days. Multisystem phenotyping, including blood biomarkers and cardiovascular and renal magnetic resonance imaging with matched computed tomography coronary and pulmonary angiography, is reported in *Table 2*. Compared with controls, the circulating concentrations of intercellular adhesion molecule 1 (ICAM-1), vascular cell adhesion molecule 1 (VCAM-1), peak C-reactive protein (CRP), and peak ferritin were increased at enrolment. Von Willebrand activity, factor VIII levels, and fibrinogen were increased in post-COVID-19 patients at enrolment, and factor VIII remained persistently high at 28–60 days post-discharge (*Table 2*).

**Table 2:** Multi-system phenotyping: serial electrocardiography, biomarkers of inflammation, metabolism, renal function, haemostasis, and heart, lung, and kidney imaging at 28–60 days post-discharge

	All	Control	COVID-19	*P*-value
*Electrocardiogram*				
*Myopericarditis criteria*				
Admission	*N* = 37	*N* = 10	*N* = 27	
	7 (19%)	0 (0%)	7 (26%)	0.155
Enrolment	*N* = 36	*N* = 10	*N* = 26	
	7 (19%)	0 (0%)	7 (27%)	0.155
28–60 days post-discharge	*N* = 35	*N* = 10	*N* = 25	
	2 (6%)	0 (0%)	2 (8%)	1.000
*CT chest 28–60 days post-discharge*				
	*N* = 35	*N* = 9	*N* = 26	
Ground glass opacity and/or consolidation	15 (43%)	0 (0%)	15 (58%)	0.004
Reticulation and/or architectural distortion	11 (31%)	0 (0%)	11 (42%)	0.033
Atelectasis	3 (9%)	0 (0%)	3 (12%)	0.553
Pulmonary arterial thrombus	1 (3%)	0 (0%)	1 (4%)	1.000
Visual estimate of % of total lung area abnormal	16.4 ± 23.9	0.0 ± 0.0	22.0 ± 25.4	0.015
*CT coronary angiogram 28–60 days post-discharge*				
	*N* = 35	*N* = 9	*N* = 26	
Coronary calcium—Agatston score	52 ± 102	16 ± 47	65 ± 114	0.224
MESA percentile	63.9 ± 25.1	41.0 ±-	65.5 ± 25.2	0.365
Obstructive coronary artery disease	4 (12%)	0 (0%)	4 (16%)	0.554
*FFR_CT_ patient-level (all coronary arteries) 28–60 days post-discharge*				
	*N* = 33	*N* = 9	*N* = 24	
Median FFR_CT_	0.92 ± 0.04	0.94 ± 0.01	0.92 ± 0.04	0.133
Minimum FFR_CT_ ≤ 0.8	11 (33%)	1 (11%)	10 (42%)	0.212
*Cardiac MRI 28–60 days post-discharge*				
	*N* = 34	*N* = 9	*N* = 25	
LV end diastolic volume index, mL/m^2^	74.0 ± 14.1	79.1 ± 14.2	72.2 ± 13.9	0.209
LV end systolic volume index, mL/m^2^	31.5 ± 9.8	31.1 ± 9.8	31.7 ± 10.0	0.891
LV ejection fraction, %	58.0 ± 7.7	61.2 ± 6.4	56.9 ± 7.9	0.153
LV mass, g	103.4 ± 29.9	124.8 ± 25.5	95.7 ± 27.9	0.010
RV end diastolic volume index, mL/m^2^	73.3 ± 15.6	85.7 ± 10.4	68.6 ± 14.7	0.003
RV end systolic volume index, mL/m^2^	31.7 ± 8.2	34.3 ± 5.6	30.8 ± 8.9	0.285
RV ejection fraction, %	55.8 ± 9.9	59.9 ± 5.6	54.3 ± 10.7	0.150
*Myocardial tissue characterization*				
	*N* = 34	*N* = 9	*N* = 25	
Increased global T1 (>1233 ms)	9 (26%)	2 (22%)	7 (28%)	1.000
Increased global T2 (>44 ms)	0 (0%)	0 (0%)	0 (0%)	1.000
T2 ratio (myocardium/serratus anterior muscle)	1.68 ± 0.22	1.64 ± 0.12	1.70 ± 0.25	0.561
Increased global extracellular volume (>27.4%)	15 (44%)	1 (11%)	14 (56%)	0.047
*Late gadolinium enhancement*				
	*N* = 35	*N* = 10	*N* = 25	
Myocardial late gadolinium enhancement	3 (9%)	0 (0%)	3 (12%)	0.549
Ischaemic distribution	1 (3%)	0 (0%)	1 (4%)	1.000
Non-ischaemic distribution	2 (6%)	0 (0%)	2 (8%)	1.000
*Myocardial inflammation (Lake Louise criteria)*				
No evidence (0/2)	13 (37%)	10 (100%)	3 (12%)	0.001
Probable (1/2)	10 (29%)	0 (0%)	10 (40%)	
Definite (2/2)	12 (34%)	0 (0%)	12 (48%)	
*Renal MRI*				
	*N* = 35	*N* = 10	*N* = 25	
Average volume of right and left kidneys, mL	159 ± 35	173 ± 32	154 ± 35	0.141
Average cortex T1 of right and left kidneys, ms	1541 ± 71	1507 ± 68	1555 ± 68	0.073
Average medulla T1 of right and left kidneys, ms	1967 ± 74	1974 ± 70	1964 ± 77	0.747
Average T1 corticomedullary differentiation of kidneys	0.78 ± 0.03	0.76 ± 0.02	0.79 ± 0.02	0.004
*Biomarkers at enrolment, central laboratory*				
	*N* = 36	*N* = 10	*N* = 26	
eGFR, mL/min/1.73 m^2^	95 (81, 102)	95 (91, 101)	93 (81, 103)	0.891
C-reactive protein, mg/L	2.8 (1.1, 5.8)	1.4 (0.9, 3.6)	3.2 (1.3, 6.7)	0.120
NT pro BNP, ng/L	80 (45, 163)	65 (42, 81)	140 (73, 219)	0.068
Total bilirubin, μmol/L	5.4 (4.1, 8.6)	10.4 (8.5, 12.6)	4.9 (3.7, 6.4)	0.003
Total cholesterol, mmol/L	5.30 ± 1.34	4.93 ± 0.97	5.44 ± 1.44	0.317
Triglycerides, mmol/L	2.68 ± 1.77	1.83 ± 1.29	3.01 ± 1.85	0.074
HDL cholesterol, mmol/L	1.11 ± 0.22	1.13 ± 0.21	1.10 ± 0.22	0.659
ICAM-1, ng/mL	503 (419, 633)	410 (384, 444)	582 (495, 685)	<0.001
VCAM-1, ng/mL	858 (692, 1164)	654 (636, 728)	981 (823, 1254)	0.002
Endothelin-1, pg/mL	2.2 (1.9, 2.7)	2.8 (2.1, 3.0)	2.1 (1.8, 2.3)	0.039
IL-6, pg/mL	3.9 (2.9, 5.8)	3.2 (1.8, 4.6)	4.6 (2.9, 6.4)	0.080
ST2, ng/mL	21.1 (17.0, 28.3)	20.0 (14.8, 24.0)	23.2 (18.2, 30.6)	0.165
p-selectin, ng/mL	69 (53, 86)	46 (38, 62)	70 (60, 86)	0.164
D-dimer, ng/mL	207 ± 221	107 ± 67	247 ± 249	0.090
Fibrinogen, g/L	3.69 ± 1.49	2.88 ± 1.00	4.01 ± 1.55	0.041
Factor VIII, IU/dL	160 ± 91	93 ± 44	187 ± 92	0.004
Antithrombin, IU/dL	110 ± 16	105 ± 16	112 ± 15	0.198
Protein S	95.1 ± 23.7	95.1 ± 30.2	95.1 ± 21.4	0.998
Protein C	130.1 ± 29.6	112.9 ± 26.5	136.9 ± 28.4	0.028
VWF: GP1bR	209 ± 124	114 ± 41	248 ± 126	0.003
VWF: Ag	209 ± 116	151 ± 51	233 ± 127	0.059
*Biomarkers at 28–60 days post-discharge, central laboratory (control group samples from enrolment visit)*				
	*N* = 37	*N* = 10	*N* = 27	
eGFR, mL/min/1.73 m^2^	94 (80, 99)	95 (91, 101)	92 (78, 97)	0.502
C-reactive protein, mg/L	1.9 (1.1, 3.3)	1.4 (0.9, 3.6)	2.0 (1.4, 3.2)	0.698
NT proBNP, ng/L	80 (61, 115)	65 (42, 81)	84 (70, 183)	0.068
D-dimer, ng/mL	137 ± 103	107 ± 67	151 ± 115	0.265
ICAM-1, ng/mL	419 (363, 503)	410 (384, 444)	450 (362, 555)	0.400
VCAM-1, ng/mL	671 (643, 953)	654 (636, 728)	841 (652, 974)	0.183
Endothelin-1, pg/mL	2.3 (2.1, 3.0)	2. 86 (2.1, 3.0)	2.3 (2.1, 2.7)	0.327
IL-6, pg/mL	2.4 (1.8, 4.4)	3.2 (1.8, 4.6)	2.4 (1.8, 4.0)	0.845
ST2, ng/mL	20.4 (14.9, 23.3)	20.0 (14.8, 24.0)	20.4 (15.2, 23.1)	0.969
p-selectin, ng/mL	59 (50, 82)	46 (38, 62)	60 (52, 82)	0.172
Prothrombin time, s	11.1 ± 0.9	11.3 ± 0.8	11.0 ± 0.9	0.321
D-dimer, ng/mL	137 ± 103	107 ± 67	151 ± 115	0.265
Fibrinogen, g/L	3.09 ± 0.90	2.88 ± 1.00	3.19 ± 0.86	0.375
Factor VIII, IU/dL	131 ± 54	93 ± 44	147 ± 50	0.005
Antithrombin, IU/dL	111 ± 17	105 ± 16	113 ± 17	0.187
Protein S	97.4 ± 22.4	95.1 ± 30.2	98.4 ± 18.8	0.701
Protein C	120.7 ± 24.3	112.9 ± 26.5	124.1 ± 23.0	0.227
VWF: GP1bR	123 ± 48	114 ± 41	127 ± 50	0.460
VWF: Ag	153 ± 60	151 ± 51	154 ± 64	0.900

Summaries are mean ± SD, median (IQR), or *N* (%). *P*-values from *t*-test, Wilcoxon–Mann–Whitney test, or Fisher's exact test. All *P*-values are two-sided. No adjustments were made for multiple comparisons.

CMR, cardiovascular magnetic resonance; CT, computed tomography; eGFR, glomerular filtration rate; ΝΤ-proBNP, N-terminal pro-brain natriuretic peptide; eGFR (CKD-EPI), estimated glomerular filtration rate using the chronic kidney disease epidemiology (CKD-EPI equation); LV, left ventricle; MESA, multi-ethnic study of atherosclerosis; NT-proBNP, N-terminal pro-B-type natriuretic peptide; RV, right ventricle; T1, longitudinal relaxation time; T2, transverse relaxation time; vWF: Ag, von Willebrand factor antigen.

^a^Categorical data are summarized as frequency and percentage and compared between groups using Fisher's exact tests. Continuous data are summarized as mean and standard deviation, or median, and interquartile range (IQR, defined as the upper and lower quartiles) and compared between groups using Kruskal–Wallis tests.

### Convalescent health status

Health status, including health-related quality of life, physical function, and aerobic exercise capacity reflected by predicted VO_2_ max mL/(kg min), in post-COVID-19 patients compared to controls (*Table 3*), was reduced at enrolment in-hospital, and these differences persisted 28–60 days post-discharge. Half of the post-COVID-19 patients were referred to the respiratory outpatient clinic during the first year post-discharge (Supplementary material online, *Table S1*).

**Table 3:** Health status, illness perception, anxiety and depression, and physical function

	All	Control	COVID-19	*P*-value
Enrolment	*N* = 37	*N* = 10	*N* = 27	
28–60 days post-discharge	*N* = 37	*N* = 10	*N* = 27	
*Health-related quality of Life, EQ-5D-5L*				
Heath utility ccore at enrolment	0.77 ± 0.25	0.85 ± 0.27	0.74 ± 0.24	0.245
Heath utility score at 28–60 days post-discharge	0.77 ± 0.28	0.85 ± 0.27	0.74 ± 0.29	0.315
Your health today VAS at enrolment	68.19 ± 24.02	85.50 ± 7.98	61.78 ± 24.88	0.006
Your health today VAS at 28–60 days post-discharge	75.54 ± 19.96	85.50 ± 7.98	71.85 ± 21.85	0.064
*Brief illness perception questionnaire score*				
At enrolment	39.1 ± 12.4	32.9 ± 12.5	41.4 ± 11.7	0.062
At 28–60 days post-discharge	35.8 ± 14.8	32.9 ± 12.5	36.8 ± 15.7	0.483
*Anxiety and depression, PHQ-4*				
Anxiety score at enrolment	1.58 ± 2.03	0.90 ± 1.91	1.85 ± 2.05	0.216
Anxiety score at 28–60 days post-discharge	1.62 ± 2.24	0.90 ± 1.91	1.89 ± 2.33	0.238
Depression score at enrolment	2.03 ± 2.08	0.80 ± 1.75	2.50 ± 2.02	0.026
Depression score at 28–60 days post-discharge	1.51 ± 2.01	0.80 ± 1.75	1.78 ± 2.06	0.193
Total score at enrolment	3.61 ± 3.87	1.70 ± 3.62	4.35 ± 3.77	0.065
Total score at 28–60 days post-discharge	3.14 ± 4.13	1.70 ± 3.62	3.67 ± 4.24	0.203
*Physical function*				
*IPAQ score at enrolment*				
Low	21 (62%)	2 (20%)	19 (79%)	0.002
Moderate	1 (3%)	1 (10%)	0 (0%)	
High	12 (35%)	7 (70%)	5 (21%)	
*IPAQ score at 28–60 days post-discharge*				
Low	16 (47%)	2 (20%)	14 (58%)	0.026
Moderate	6 (18%)	1 (10%)	5 (21%)	
High	12 (35%)	7 (70%)	5 (21%)	
DASI score at enrolment	24.9 ± 21.1	48.0 ± 15.7	16.0 ± 15.5	<0.001
DASI score at 28–60 days post-discharge	28.3 ± 20.8	48.0 ± 15.7	21.1 ± 17.6	0.001
VO_2_ max estimated at enrolment, mL/(kg min)	20.3 ± 9.1	30.3 ± 6.7	16.5 ± 6.7	<0.001
VO_2_ max estimated at 28–60 days post-discharge, mL/(kg min)	21.8 ± 8.9	30.3 ± 6.7	18.7 ± 7.6	0.001

Summaries are mean ± SD, median (IQR), or *N* (%). *P*-values from *t*-test, Wilcoxon–Mann–Whitney test, or Fisher's exact test.

### Human vascular pharmacology

**Figure 1:**
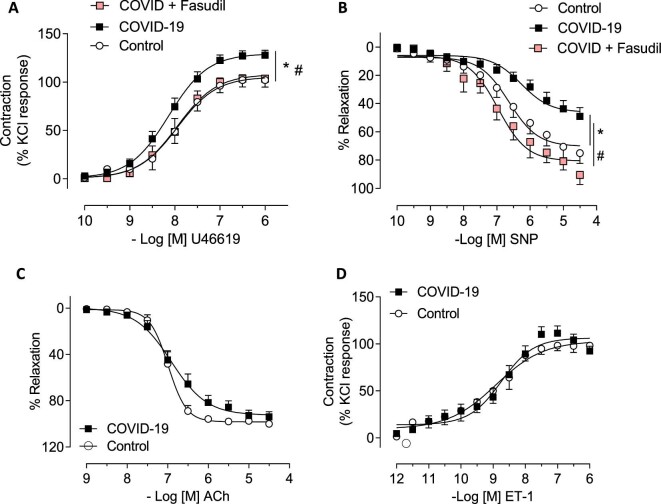
Vascular reactivity of gluteal biopsies-isolated small vessels from control and COVID-19 patients. Cumulative concentration–response curves to (*A*) U46619 (a thromboxane A2 analogue) (control *n* = 8; COVID-19 *n* = 24) (*P* = 0.012) and (*B*) sodium nitroprusside (endothelium-independent vasodilator) (control *n* = 8; COVID-19 *n* = 18) (*P* = 0.039) in the presence of fasudil (Rho-kinase inhibitor; 1 µmol/L; 30 min; *n* = 5–6). Concentration–response curves to (*C*) acetylcholine (endothelium-dependent vasodilator) (control *n* = 4; COVID-19 *n* = 13) (*P* = 0.631) and (*D*) ET-1 (control *n* = 6; COVID-19 *n* = 17) (*P* = 0.880) in small arteries isolated from gluteal biopsies derived from control and COVID-19 patients. Relaxant responses were expressed as percentage of U46619-induced pre-constriction and contraction as percentage of KCl responses. Results are expressed as mean ± SEM. * vs. control; # vs. COVID-19.

The median (IQR) time from COVID-19 diagnosis based on a PCR-positive result to the date of the gluteal biopsy was 160 (138, 212) days. Compared to contractile responses to U46619, the thromboxane A2 agonist, in small arteries isolated from control patients, the contractile responses in small arteries isolated from post-COVID-19 patients were increased (*P* = 0.039) (*Figure 1**A*). SNP-induced vasorelaxation (endothelium-independent) was significantly reduced relative to controls (*P* = 0.012) (*Figure 1**B*). There were no between-group differences in acetylcholine (ACh)-induced vasorelaxation (endothelium-dependent) (*P* = 0.880) (*Figure 1**C*) or contractile responses to ET-1 (*P* = 0.631) (*Figure 1**D*). A sensitivity analysis excluding data from patients with a history of hypertension was also performed. The CCRC responses to U46619 and SNP were not altered when adjusting for controls vs. COVID-19 participants with a history of hypertension (U46619 *E*_max_ including patients with a history of hypertension: 102.1% vs. 127.9%, *P* = 0.012; U46619 *E*_max_ excluding patients with a history of hypertension: 106.8% vs. 134.5%, *P* = 0.043; SNP *E*_max_ including patients with a history of hypertension: 72.1% vs. 49.0%, *P* = 0.039; SNP *E*_max_ excluding patients with a history of hypertension: 74.3% vs. 47.4%, *P* = 0.043) (Supplementary material online, *Figure S2A, B*). In subsequent studies, selective pre-treatment of the small arteries with fasudil, a potent inhibitor of the Rho-kinases ROCK1 and ROCK2, restored SNP-mediated vasodilatation and U46619 hypercontractile response (*Figure 1**A, B*). Fasudil did not change SNP and U46619 responses in small arteries from control patients (Supplementary material online, *Figure S3A, B*).

### Histopathology and immunohistochemistry

Aniline blue and picrosirius red stains were used to assess collagen distribution within the small arteries (Supplementary material online, *Figure S4A, B*). The mean tissue proportion (Supplementary material online, *Figure S4A*) of aniline blue stain uptake in COVID-19 samples was 69.7% (95% CI: 67.8–71.7), equating to a mean difference of 4.9% (95% CI: 0.7–9.0) compared with control samples, which was 64.9% (95% CI: 59.4–70.3) (*P* = 0.029). The mean tissue proportion (Supplementary material online, *Figure S4B*) of picrosirius red stain uptake in COVID-19 samples was 68.6% (95% CI: 64.4–72.8) with a mean difference of 8.5% (95% CI: 1.0–16.0) compared with 60.1% uptake in controls (95% CI: 55.4–64.8) (*P* = 0.028).

**Figure 2:**
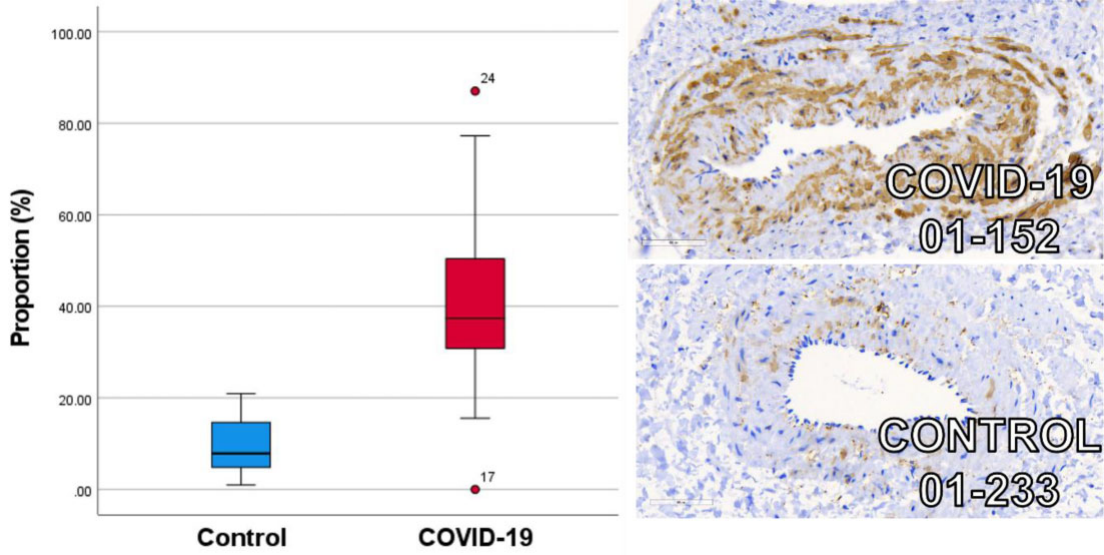
Box and whisker plot of COVID-19 (red) and age, sex, and cardiovascular risk factor matched controls (blue) immunohistochemical staining for phosphorylated myosin light chain antibody uptake. Increased mean proportion of staining for phosphorylated myosin light chain antibody uptake is observed in patients following COVID-19 (*n* = 21) (40.1%; 95% CI: 30.9–49.3) compared with age, sex, and cardiovascular risk factor matched controls (*n* = 9) (10.0%; 95% CI: 4.4–15.6) (*P* < 0.001). Illustrative example images are provided demonstrating the ×40.0 magnification with 60 uM scale bar.

Immunohistochemical analysis (*Figure 2*) was undertaken to investigate the downstream effects of Rho-kinase in this cohort, with staining for phosphorylated myosin light chain (pMLC) antibodies (Abcam ab2480). pMLC phosphorylation reflects enhanced sensitivity towards Ca^2+^ linking actin–myosin filaments for VSMC contraction. Overall, much greater pMLC antibody positive tissue was observed in the COVID-19 vessels at ×40.0 magnification, using a total slide tissue proportion threshold analysis technique, COVID-19 (40.1%; 95% CI: 30.9–49.3; *n* = 21) vs. controls (10.0%; 95% CI: 4.4–15.6; *n* = 9) *P* < 0.001. This mechanism for Rho-kinase inhibition, through a reduction in MLC activity, corroborates the mediation of hypercontractility and impaired vasodilation observed during wire myography experiments.

### Whole transcriptome analysis in intact arteries

**Figure 3:**
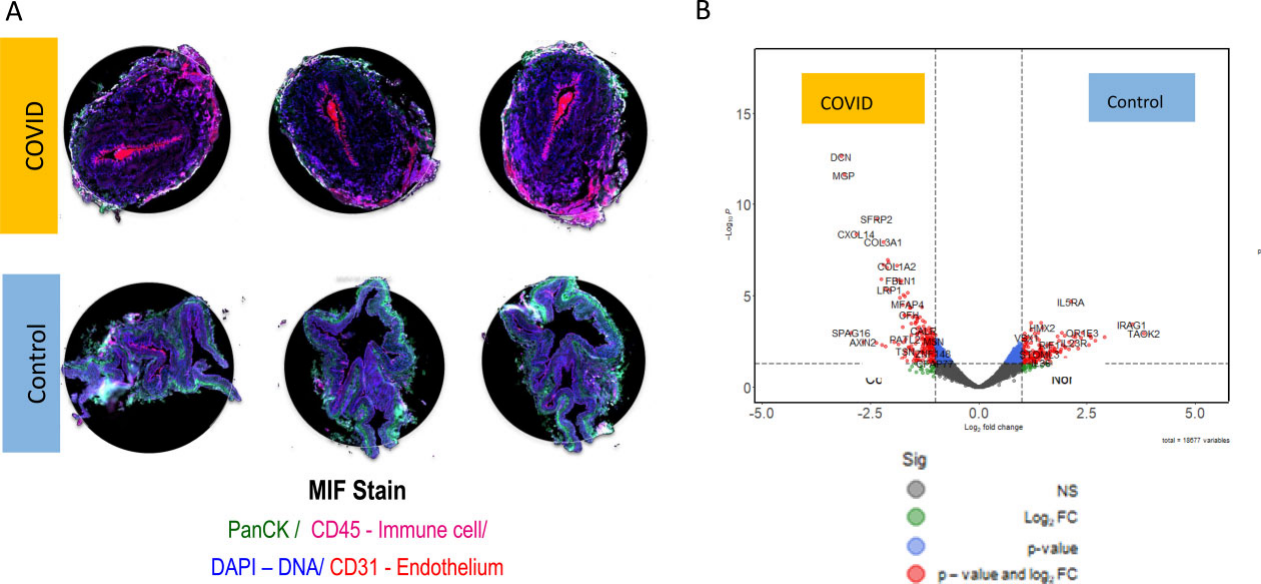

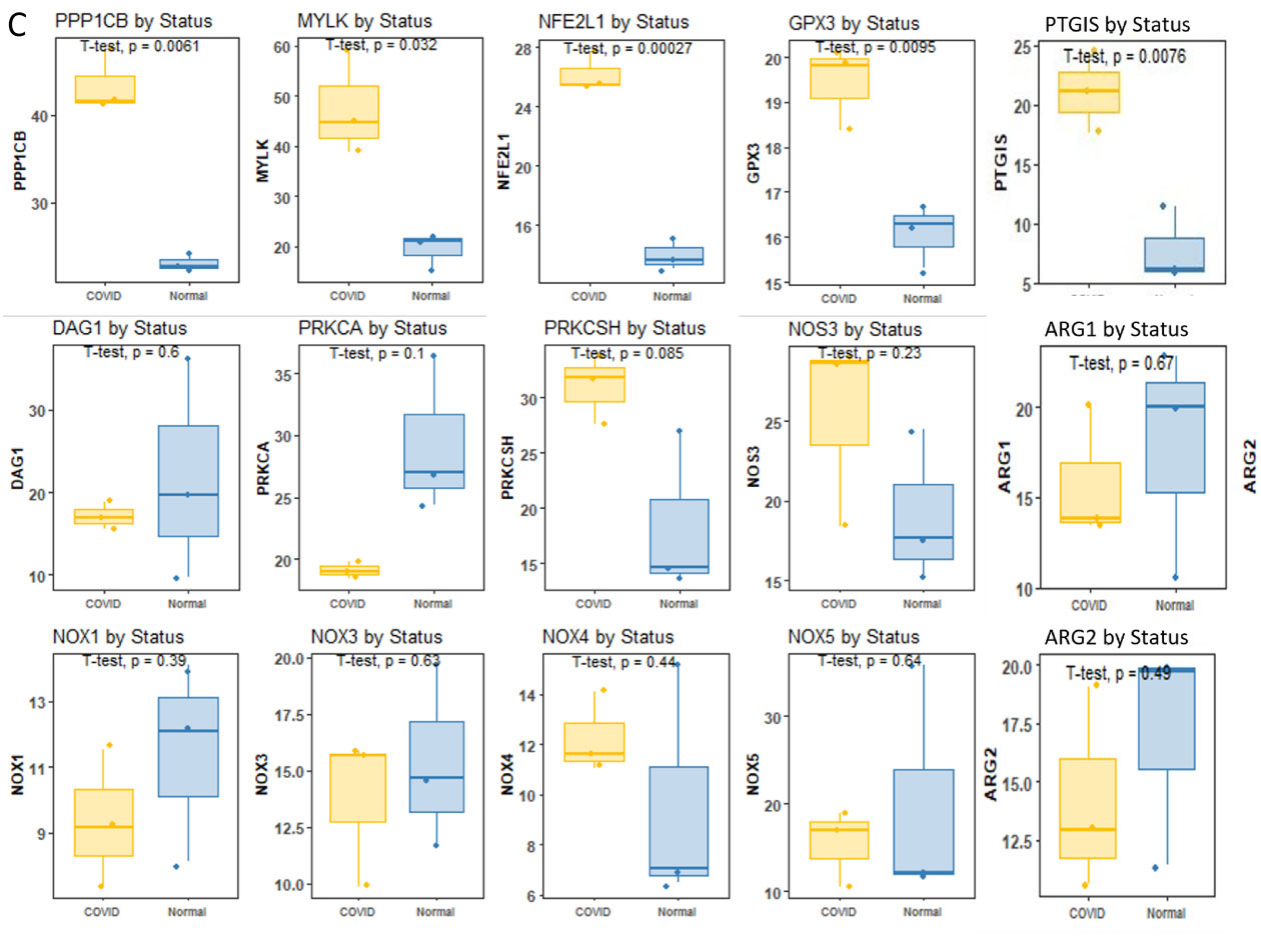

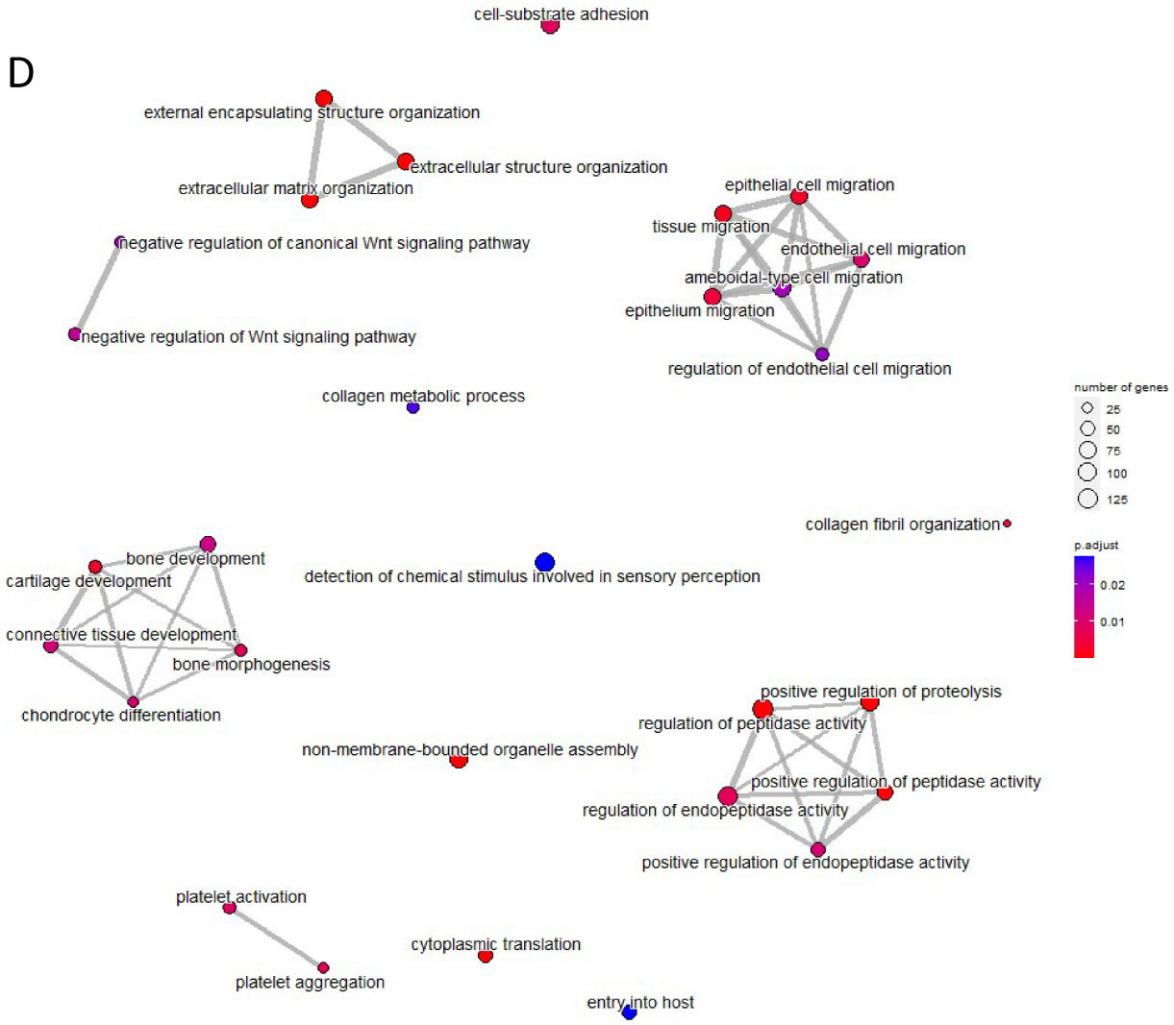
Summary of whole vascular transcriptomics analysis by technique: (*A*) Merthiolate-iodine-formaldehyde staining of paraffin-embedded small artery sections, including three sections from a patient 3 months after COVID-19 compared with three sections from an age, sex, and cardiovascular risk factor matched control; (*B*) volcano plot of gene expression from a patient post-COVID-19 compared with an age, sex, and cardiovascular risk factor matched control; (*C*) gene expression of downstream effectors of vascular function in a patient following COVID-19 compared with an age, sex, and cardiovascular risk factor matched control. In addition to the displayed boxplot results, there were no differences in gene expression between vessel sections for DAG1, MYL2, PPP1R12A, PPP1CA, NOS3, NOX1, NOX3, NOX4, NOX5, NOXO1, NOXA1, DUOX1, DUOX2, ARG1, CYBA, NCF1, NFE2, NFE2L1, NFE2L2, NFE2L3, SOD1, SOD2, SOD3, GPX1, GPX4, GPX5, GPX6, GPX7, GPX8, HMOX1, HMOX2, PRDX1, PRDX2, PRDX4, PRDX5, PRDX6, GUCY1B1, GCK, GCKR, PTS, PCBD2, PCBD1, GCHFR, and SPR. (*D*) Gene ontology gene sequence expression analysis results organized by biological relevance in a patient following COVID-19 compared with an age, sex, and cardiovascular risk factor matched SARS-CoV-2 serology-negative control patient.

Proof-of-concept studies of gene expression spatial profiling in intact arteries, involving two sample sets, one from a post-COVID-19 patient and one from a control with similar age, sex, and cardiovascular risk factors, were undertaken. The clinical characteristics of whom are summarized as vignettes within the supplement. Three paraffin-embedded vascular sections from each patient underwent whole transcriptome analysis (*Figure 3**A*). Gene pathways involving extracellular matrix (ECM) regulation and ECM proteoglycans were observed in the COVID-19 arteries (Supplementary material online, *Figure S5*). Spatial deconvolution revealed enhanced infiltration of fibroblasts and macrophages and depletion of natural killer cell populations (Supplementary material online, *Figure S6*). The DCN gene responsible for decorin synthesis appeared upregulated in COVID-19 patients (*Figure 3**B*). The matrix gla protein gene was upregulated, which implicates alterations in calcium ion homeostasis and the ECM (Supplementary material online, *Figure S3B*).

To evaluate the regulation of downstream effectors involved in vascular homeostasis, further gene-specific analysis was undertaken, including diacylglycerol (DAG), proteinase K (PKC) and PKC substrate, myosin phosphatases, myosin light chain kinases endothelial nitric oxide synthase, and prostacyclin synthase (PTGIS; *Figure 3**C*). The bioinformatics case-control analysis revealed increases in myosin light chain kinase expression (*P* = 0.032) and PTGIS (*P* = 0.008), and trends towards a reduction in PKC (*P* = 0.100) and an increase in PKC substrate (*P* = 0.09). Nuclear factor erythroid-2 like 1 (NFE2L1) and glutathione peroxidase 3, which regulate oxidative stress and inflammatory responses, were upregulated following COVID-19 (*P* < 0.001 and *P* = 0.010, respectively). In addition, gene ontology gene sequence expression analysis revealed evidence of upregulated viral replication mechanisms, platelet activation, connective tissue organization, and tissue morphogenesis (*Figure 3**D*).

## Discussion

This study has provided novel insights into the vascular mechanisms of COVID-19 in convalescent hospitalized patients with persisting symptoms and impaired aerobic exercise capacity. We took care to match control patients with cardiovascular risk factors and co-morbidity to adjust for disease processes associated with impaired vascular function.

In laboratory studies, compared with controls, small peripheral arteries isolated from post-COVID-19 patients exhibited enhanced vasoconstriction and impaired endothelium-independent vasodilation restored in the presence of fasudil. A case-control experiment also observed changes in VSMC Ca^2+^ homeostasis and Rho-kinase activation. There were no differences in endothelial function between patients recovering from post-COVID-19 and control patients. This finding is likely to be explained by the high prevalence of cardiovascular risk factors in the control group and the lack of ACE2 expression on endothelial cells.^11,23,24^ By contrast, VSMCs exhibit basal expression of ACE2 implicating direct viral involvement with the VSMC membrane.^25^ Biomarkers of vascular inflammation including ET-1, ICAM-1, and VCAM-1 with factor VIII, protein C, and vWF activity were increased at enrolment, although returned to comparable levels with controls within 60 days of discharge from hospital, suggesting that persistent acute inflammation is not responsible for post-COVID-19. Our findings, therefore, suggest a post-inflammatory syndrome including fibrosis, inappropriate Rho-kinase activation, and direct viral invasion with resultant VSMC damage as mechanisms for an endothelial-independent post-COVID-19 vascular dysfunction. Rho-kinase inhibition appears to be a novel target for improving vascular function in patients with cardiovascular symptoms after COVID-19 (long COVID).

The impairment in vasorelaxation to SNP, a direct NO donor, and enhanced vasoconstriction induced by U46619 (a thromboxane A2 analogue), implicate abnormalities in the VSMC located within the tunica media.^26^ Calcium ions are second messengers in mammalian cells and participate in the regulation of vascular tone. An increase in cytosolic Ca^2+^ stimulates VSMC contraction.^27^

Our findings extend those of Gustafson *et al*., who observed endothelial dysfunction during the acute phase of COVID-19 similar to disease-matched control patients.^28^ In our primary cohort, involving 159 hospitalized patients, circulating concentrations of ICAM-1, VCAM-1, and factor VIII remained elevated at 28–60 days, whereas overall, CRP and ferritin were not.^4^ In the cohort described in this study, factor VIII remained increased, reflecting endothelial activation^29^ persisting after the resolution of the acute illness, and impairments in physical function and aerobic exercise capacity in patients following COVID-19 were reported in keeping with post-COVID-19 conditions. ICAM-1, VCAM-1, and altered proteoglycan barrier function cause increased endothelial permeability facilitating macrophage recruitment into the vascular wall,^30,31^ reflecting the profibrotic effects of vascular inflammation^32^ observed in our study (Supplementary material online, *Figure S4A, B*). Novel histopathological evidence of vascular collagen deposition in our post-COVID-19 patients and whole transcriptome upregulation of extracellular membrane gene pathways implicate vascular fibrosis and VSMC dysfunction as the mechanisms of persisting cardiovascular symptoms and impaired aerobic capacity in post-COVID-19 conditions. Furthermore, the increase in pMLC, reflecting enhanced Ca^2+^ sensitivity in VSMC from post-COVID-19 patients, supports the results of the wire myography experiments and related conclusion that VSMC dysfunction is implicated following COVID-19. The downstream observation of increased pMLC is also in keeping with increased Rho-kinase activity and complements the amelioration of inappropriate vasoconstriction and impaired vasodilation by the Rho-kinase inhibitor, fasudil.

Prior studies have evidenced impaired endothelium-dependent flow-mediated dilation,^33^ but a control group was lacking. Other studies have focused on the role of Rho-kinase in mediating endothelial cell glycocalyx disruption in acute COVID-19^34^ (but not post-COVID-19 conditions), and molecular therapies that preserve endothelial cell connections may be beneficial in acute COVID-19.^35^ Our study examined vascular mechanisms in patients with post-COVID-19 conditions vs. controls matched for age, sex, and cardiovascular morbidity. The objective was to identify tractable targets for therapy development towards improving cardiovascular symptoms in future clinical trials. This mechanistic study was not designed or powered to provide correlations between vascular function and clinical disease markers. Convalescent patients post-COVID-19 had multisystem evidence of cardiovascular involvement and impaired aerobic exercise capacity, compared with matched controls. To the best of our knowledge, our study is the first to perform invasive biopsies in patients with post-COVID-19 conditions to examine the mechanisms of vascular dysfunction using intact human arteries. The inclusion of carefully selected controls enabled a case-control design to discriminate pathways that are distinctly associated with COVID-19, as opposed to vascular risk factors. Flow-mediation dilation experiments incorporating brachial artery occlusion release have also been described in post-COVID-19 patients.^36–38^ However, brachial artery flow-mediated dilatation has some technical limitations, and non-endothelial-dependent pathways are difficult to assess *in vivo*.^39^ Our study adds novel laboratory mechanistic data implicating vascular fibrosis, VSMC dysfunction, and Rho-kinase pathway activation in the cardiovascular contribution to post-COVID-19 conditions.

Physiological Rho-kinase agonists mediate vascular inflammation, which include cell-adhesion molecules, ET-1, platelet-derived growth factor, sphingosine-1-phosphate, shear, and mechanical stress.^40,41^ Interestingly, patients with Bartter's or Gitelman's syndrome have increased ACE2 and blunted Rho-kinase responses, and these patients appear to have an innate resistance to SARS-CoV-2 infection.^42,43^

Rho-kinase signalling is implicated in abnormal vasomotor tone.^44^ Nitric oxide dilates resistance arteries by activating the myosin light chain phosphatase (MLCP) in a cGMP-dependent manner, thereby reducing the apparent Ca^2+^ sensitivity of the contractile apparatus. MLCP inactivation via the RhoA/Rho-kinase pathway antagonizes this Ca^2+^-desensitizing effect that, in turn, can be restored using RhoA/Rho-kinase inhibitors.^45^ In our study, pre-treatment of small arteries with fasudil restored SNP-mediated vasodilatation. The endothelium in small resistance arteries induces relaxation via NO production and via the production of additional factors, including endothelium-derived hyperpolarizing factor and prostacyclin. In our post-COVID-19 case-control design, we observed impaired SNP-mediated vasodilation post-COVID-19 but no alteration in ACh-induced vasorelaxation when compared to matched controls with a similar extent of cardiovascular disease during wire myography cumulative concentration–response experiments. In patients with post-COVID-19 conditions, we demonstrate that NO signalling in VSMCs (independent of endothelial cells) and VSMC-mediated vasorelaxation is deficient, since SNP is a NO donor. On the other hand, endothelium-dependent pathways, including prostacyclin, may be preserved, and we did observe upregulation of prostacyclin synthase in VSMC transcriptomics. The post-COVID-19 arteries were both hypercontractile and less responsive to NO-induced relaxation; these effects were ameliorated by treatment with fasudil.

The case-control design is a strength of our study. The pre-specified matching of controls with similar age, sex, and cardiovascular risk factors and disease explains why small artery endothelial function was similar in the post-COVID-19 patients and controls. Specifically, the eligibility criteria for controls required a history of cardiovascular risk factors, in addition to careful age and sex matching and non-COVID-19 morbidity. The presence and extent of left ventricular hypertrophy and coronary artery disease revealed by MRI and CT coronary angiography, respectively, were similar in the controls compared to the patients (*Table 2*). Based on the objective measurements of cardiovascular disease in the controls, evidence of endothelial dysfunction would be expected.

Spatial transcriptomics provided insights into the mechanisms of post-COVID-19 small artery dysfunction. The transcriptomics was intended to spatially probe gene expression pathways implicated in vascular function, and given the findings from small artery myography, we specifically probed endothelial-independent VSMC pathways. Although we did not observe any changes in oxidative stress-associated genes at this stage, we did observe alterations in the VSMC myosin phosphatase pathway and a trend towards alterations in PKC, and these pathways are known to regulate vascular tone. These proof-of-concept findings merit further investigation in a larger number of patients.

### Limitations

Although the sample size is modest, to the best of our knowledge, it is larger than previous mechanistic human studies. This study was designed to have sufficient statistical power to identify or exclude a difference in maximum small artery contraction between patients previously hospitalized with COVID-19 and cardiovascular risk factor matched controls. Further studies are warranted to validate the relationship between vascular dysfunction following COVID-19 and the development of post-COVID-19 conditions. Since our patients were unvaccinated, the results are most relevant to unvaccinated individuals. The intracellular mechanisms of alterations in SNP-mediated vasorelaxation merit further study. The data using spatial transcriptomics and VSMC are exploratory, and future studies would be required to validate our findings.

## Conclusion

In summary, compared to controls, vascular fibrosis is enhanced in small arteries from patients with post-COVID-19 conditions. These arteries are hypercontractile and exhibit impaired non-endothelial-dependent vasorelaxation, and these responses improve following treatment with fasudil, implicating Rho-kinase activation as a therapeutic target.

Rho-kinase inhibitors are indicated treatments for cerebral vasospasm and glaucoma,^16,46^ and have emerging potential for use in other vascular conditions (e.g. erectile dysfunction, migraine)^47,48^ and chronic airway disease.^49^ Our novel findings suggest that RhoA/Rho-kinase signalling is a therapeutic target in patients with cardiovascular symptoms post-COVID-19. Given that Rho-kinase inhibitors are clinically available but not licensed for use in post-COVID-19 conditions, randomized, controlled trials seem warranted.

## Supplementary Material

pvad025_Supplemental_File

## The CISCO-19 Investigators

Chief Investigator: Colin Berry BSc/MBChB/PhD.

## Site investigators

Queen Elizabeth University Hospital.

Kenneth Mangion MBChB/PhD, Pauline Hall Barrientos PhD, Kevin G. Blyth MBChB/PhD, Michael Briscoe MBChB, Colin Church MBChB/PhD, Christian Delles MBChB/PhD, Stephen Dobbin MBChB, Keith Gillis MBChB/PhD, Antonia Ho MBChB/PhD, Anna Kamdar BMedSci, David J. Lowe MBChB/MD, Kaitlin J. Mayne MBChB, Patrick B. Mark MBChB/PhD, Christopher McGinley MBChB, Connor McKee BMedSci/MBChB, Andrew Morrow MBChB, Oliver Peck MBChB, Alastair J. Rankin BSc/MBChB, Giles Roditi MBChB, Claire Rooney MBChB/PhD, Sarah A. Spiers PhD, David Stobo MBChB, Robert Sykes MBChB/BMedSci, Ryan Wereski MBChB, Sylvia Wright MBChB/PhD, and Colin Berry BSc/MBChB/PhD.

## Royal Alexandra Hospital

Lynn Abel BN and Douglas Grieve MBChB/PhD.

## Glasgow Royal Infirmary

Catherine Bagot MBBS/MD, Hannah Bayes MBChB/PhD, Jaclyn Carberry MBChB/BMedSci, Daniel Doherty MBChB, Ian Fergus BSc, Vivienne B. Gibson PhD, Fraser Goldie MBChB, Laura Knox MBChB, Giles Roditi MBChB, Katherine Scott MBChB, David Stobo MBChB, Varun Sharma MBChB, and Robert Sykes MBChB/BMedSci.

## Glasgow Clinical Research Facility and Study Management Group

Ammani Brown BN, Andrew Dougherty BN, Kirsty Fallon BN, and Lesley Gilmour BN.

Chloe Cowan BN, Lynsey Gillespie PhD, Sharon Kean, Jurgen Van-Melckebeke BSc, Kim Moran-Jones PhD, Debra Stuart PhD, and Maureen Travers PhD.

## Glasgow Clinical Research Imaging Facility, Institute of Clinical Excellence, Queen Elizabeth University Hospital

Tracey Hopkins DCR/BSc(Hons)/PGd, Laura Dymock BSc(Hons), Evonne McLennan BSc(Hons)/PGc, Rosemary Woodward BSc/PGc, Fiona Savage BSc(Hons)/PGc, and Nicola Tynan BSc(Hons)/PGc.

## Radiology, NHS Greater Glasgow and Clyde Health Board

Sau Lee Chang MBChB, Mhairi Dupre MBChB, Lindsey Norton MBChB, Liam Peng MBChB, Giles Roditi MBChB, and David Stobo MBChB.

## Laboratory Medicine and Biorepository, NHS Greater Glasgow and Clyde Health Board

Clare Orange BSc and Rory Gunson PhD.

## Medical Physics, NHS Greater Glasgow and Clyde

Sarah Allwood-Spiers PhD, George Bruce BSc, Rosario Gonzalez-Lopez PhD, Pauline Hall Barrientos PhD, and Rebecca Stace BSc.

## University of Glasgow

School of Cardiovascular and Metabolic Health

Colin Berry MBChB/PhD, Elaine Butler BSc, Christian Delles MBChB/PhD, Jennifer S Lees MBChB/PhD, Kenneth Mangion MD/PhD, Patrick Mark MBChB/PhD, Andrew Morrow MBChB, Naveed Sattar MBChB/PhD, Robert Sykes MBChB/BMedSci, Rhian M. Touyz MD/PhD, and Paul Welsh PhD.

## Institute of Infection, Immunity and Inflammation

MRC Centre for Virology Research

Antonia Ho MBChB/PhD and Massimo Palmarini PhD.

## Institute of Health and Wellbeing

Robertson Centre for Biostatistics

John G.F. Cleland MBChB/PhD, Sharon Kean, Bernard Kelly BSc, Alex McConnachie PhD, Alasdair McIntosh PhD, Dionne Russell BSc, and Sarah Weeden PhD.

## Electrocardiology Core Laboratory

Peter W. Macfarlane DSc, Louise Inglis BSc(Hons), Jean Watt, Kathryn McLaren, and Shahid Latif MAppSci (now deceased).

## Departments of Mathematics and Statistics

Nick Hill PhD, Dirk Husmeier PhD, and Xiaoyu Luo PhD.

## National Institutes of Health

Peter Kellman PhD and Hui Xue PhD.

## HeartFlow

Amy Collinsworth MS, Sarah Mullen MBT, and Campbell Rogers MD.

## Clinical Event Committee

Heerajnarain Bulluck MBChB/PhD, David Carrick MBChB/PhD, David Corcoran MBChB/PhD, Iain Findlay MBChB/MD, Ninian N. Lang, MBChB/PhD, Vera Lennie MBChB, Ross McGeoch MD, Sabrina Nordin MBChB/PhD, Alexander Payne MBChB, Nicola Ryan MBBCh, Gruschen Veldtman MBChB/PhD, Robin P. Weir MBChB/PhD, and Stuart Watkins MBChB/MD.

Coordinators—Andrew Morrow MBChB and Robert Sykes MBChB/BMedSci.

## Steering Committee

College of Medical, Veterinary and Life Sciences, University of Glasgow—Neil Basu MD/PhD, Iain McInnes MBChB/PhD, and Stefan Siebert MD/PhD.

## Data availability

All data are incorporated into the article and its online supplementary material.
