# Supplementary material for: Vascular mechanisms of post-COVID-19 conditions: Rho-kinase is a novel target for therapy
Source: Eur Heart J Cardiovasc Pharmacother. 2023 Apr 5;9(4):371–86. doi: 10.1093/ehjcvp/pvad025 (PMC10236521; doi:10.1093/ehjcvp/pvad025)
Supplement: pvad025_Supplemental_File [file pvad025_supplemental_file.docx]

# Supplement

# Title: Vascular mechanisms of post-COVID-19 conditions: Rho-kinase activation is a novel target for therapy

Authors: Robert A. Sykes*^1,2^ BMedSci/MBChB, Karla B. Neves*^1,2^ PhD, Rhéure Alves-Lopes^1^ PhD, Ilaria Caputo^4^ BSc/MSc, Kirsty Fallon^5^ BSc Nigel B. Jamieson^6^ MBChB/PhD, Anna Kamdar BMedSci^1^, Assya Legrini^6^ BSc/MSc, Holly Leslie^6^ BSc/MRes, Alasdair McIntosh^7^ PhD, Alex McConnachie PhD^7^, Andrew Morrow^1,2^ MB BCh, Richard Macfarlane^1^ MBChB, Kenneth Mangion^1,9^ MBChB/PhD, John McAbney^8^, Augusto C. Montezano^1,10^ PhD, Rhian Touyz^1,10^ MBBCh/PhD, Colin Wood^6^ MBChB, Colin Berry ^1,2,9^ BSc/MBChB/PhD

*Joint lead authors

# Methods

## Study approval

Ethical approvals for the CISCO-19 study and vascular biology sub-study were obtained from the UK National Research Ethics Service (Reference 20/NS/0066). Informed written consent and continued eligibility assessment were obtained before conducting study procedures**.**

## **Patient recruitment and clinical features**

We undertook a prospective, observational, multicentre, secondary care cohort study assessing the prevalence and clinical significance of multiorgan injury in survivors of COVID-19 during convalescence^1,2^. We prespecified a study of vascular mechanisms of post-COVID-19 conditions using laboratory science techniques including wire myography, histopathology, cell assays and spatial transcriptomics.

## Setting

The main study involved three hospitals in the West of Scotland (population 2.2 million) – the Queen Elizabeth University Hospital, the Glasgow Royal Infirmary, and the Royal Alexandra Hospital in Paisley.

## Participant identification

Patients who received hospital care for COVID-19, with or without admission, and were alive, were prospectively screened in real-time using an electronic healthcare information system (TrakCare®, InterSystems®, USA) and daily hospital reports identifying inpatients with laboratory-positive results for COVID-19.

## Eligibility criteria

The inclusion criteria were: (1) age >18 years old; (2) history of an unplanned hospital visit, e.g., emergency department or hospitalisation>24 hours for COVID-19, confirmed by polymerase chain reaction nasopharyngeal; (3) ability to comply with study procedures; (4) ability to provide written informed consent. Accredited radiologists reported the radiology results according to current national guidelines ^3^.

The exclusion criteria were: (1) contra-indication to cardiovascular magnetic resonance imaging, e.g., severe claustrophobia, metallic foreign body; and (2) lack of written informed consent.

## Screening

A screening log was prospectively completed. The reasons for being ineligible, including lack of inclusion criteria or presence of exclusion criteria, were recorded.

## Diagnosis of COVID-19

A diagnosis of COVID-19 was based on either laboratory evidence of SARS-CoV-2 infection using a polymerase chain reaction (PCR) test on a biospecimen^4^. The laboratory tests included the Roche Cobas 6800 or Seegene SARS-CoV-2 PCR tests.

## Research schedule

The protocol involved an enrolment visit, a research imaging visit, and an optional vascular sub-study visit. The enrolment visit involved informed consent and baseline assessments during the initial hospitalisation or as soon as possible after discharge. The research imaging visit occurred 28–60 days post-discharge. This window reflected the convalescent phase and gave sufficient scope to schedule the patients.

The procedures involved the collection of clinical data and undertaking research investigations. Clinical data included demographics, medical and cardiovascular history, findings from clinical examinations, laboratory and radiological tests, cardiology tests (including an electrocardiogram (ECG) and an echocardiogram if available) and treatment. The research investigations at both visits included blood and urine samples, a 12-lead digital ECG (Beneheart R3, Mindray, Huntingdon, UK), health status questionnaires, and assessments of adverse events (Supplement). Heart, lung and kidney imaging: chest CT, pulmonary and coronary angiography, and cardiovascular and renal MRI were acquired at the second visit.

Participating patients were invited to undergo a gluteal biopsy at a third visit to obtain small arteries for in vitro studies ex vivo. This was undertaken beyond the acute phase of illness to assess for persistent vascular abnormalities following COVID-19.

Control patients who had received secondary care and had similar age, sex, and cardiovascular morbidities were prospectively screened and invited to participate. They were confirmed to be COVID-19 antibody-negative using the Roche^®^ Elecsys anti-SARS-CoV-2 S quantitative assay without previous positive polymerase chain reaction (PCR) positivity or a clinical syndrome in keeping with potential COVID-19.

## Biomarkers

Blood and urine samples were collected at enrolment (Visit 1) and 28 – 60 days post-discharge (Visit 2). The measurements were undertaken in a central laboratory, blinded to the other clinical data.

Blood samples collected into 0.109M sodium citrate (for hemostasis assays) or EDTA (for other biomarkers) were handled according to a sample handling manual provided to all sites. The blood samples were centrifuged locally, and the plasma was separated and frozen at -80ºC within 2 hours of sampling. Residual samples were transferred to the NHS Glasgow Biorepository for storage at the end of the study.

## Biochemical markers

Circulating biomarkers of inflammation (CRP, ferritin, IL-6, ST2), cardiac injury (high sensitivity cardiac troponin I, NTproBNP), lipids, endothelial proteins (ICAM-1 VCAM-1 endothelin-1, p-selectin), hemostasis (coagulation screen, Clauss Fibrinogen, D-Dimer, FVIII (one-stage), VWF Antigen and VWF: GP1bR, antithrombin, protein C, and free protein S), and renal function (serum creatinine, estimated GFR estimated using the Chronic Kidney Disease Epidemiology (CKD-EPI) equation^5^) and urinary albumin: creatinine ratio), and their changes over time were investigated.

EDTA plasma samples were stored at -80ºC in the Glasgow Biorepository until batch analysis at the end of the study. The biochemical analyses were performed in the British Heart Foundation Glasgow Cardiovascular Research Centre. EDTA plasma samples were stored to analyse high-sensitivity cardiac troponin I and NT-proBNP on the first thaw. Troponin I (ng/ml) and NT-proBNP (pg/ml) were measured in blood samples collected at Visit 1 and Visit 2. NT-pro BNP (pg/ml) was measured to provide a biochemical measurement of left ventricular remodelling (within-subject change in NT-proBNP at follow-up from baseline)^6^ and troponin I to provide a biochemical measurement of myocardial necrosis.

To measure NT-proBNP, we used an automated method (i1000SR ARCHITECT, Abbott Diagnostics, United Kingdom) calibrated and quality controlled using the manufacturer's reagents. We also participated in the National External Quality Assurance Scheme (NEQAS).

For NT-proBNP, the coefficient of variation was 3.6% and 5.5% for control materials with a mean NT-proBNP level of 5141 pg/ml and 139 pg/ml, respectively. The NT-proBNP results were provided to the Robertson Centre for Biostatistics, University of Glasgow.

## Haemostasis markers

### Sample Handling

All sodium citrate plasma samples were processed in a non-standard manner using anonymised bar-coded samples by a trained member of staff within the Glasgow Biorepository. Frozen plasma samples were transported on dry ice for central laboratory analysis in the Department of Haematology, Macewen Building, Castle Street, Glasgow Royal Infirmary, G4 0SF. The United Kingdom Accreditation Service accredits this laboratory. Plasma samples were stored at -80ºC until analysis, with residual samples being transferred to the Glasgow Biorepository for storage at the end of the study.

### Assays

All haemostasis laboratory assays were carried out using Werfen reagents on Werfen ACL TOP 550/750 or Werfen ACL AcuStar (VWF: GP1bR only) analysers, per manufacturer's guidelines using a single lot of Werfen reagent (Werfen UK, Warrington Cheshire, WA3 6DE). The coagulation screen consisted of a Prothrombin Time (PT) assay, Activated Partial Prothrombin Time (APTT) assay, Thrombin Clotting Time (TCT) assay and Fibrinogen Clauss assay with normal reference ranges of 9 - 13 seconds, 27 - 36 seconds, 11 - 15 seconds, and 1.7 – 4 g/L, respectively (all internally derived). The Fibrin D-Dimer assay (latex immunoassay) had a reference range <230 ng/ml (manufacturer derived). The one-stage FVIII assay was carried out using SynthASil reagent (Werfen, UK) and had a range of 58 – 152 IU/dL. The VWF: Ag (latex immunoassay) and VWF: GP1bR activity assay (chemiluminescent immunoassay) had reference ranges of 51 – 170 IU/dL and 52 – 172 IU/dL, respectively (internally derived). Antithrombin activity (chromogenic), Free-protein S (latex immunoassay) and Protein C activity assay (chromogenic) had reference ranges of 82 – 123 IU/dL, 75 – 137 IU/dL and 71 – 146 IU/dL, respectively (all internally derived). Haemostasis laboratory assays were completed following the fulfilment of internal quality control checks using control material traceable to International Standards, per standard laboratory operating procedures. Furthermore, all methodology used for this study is regularly subject to external quality control checks through the internationally recognised scheme, UKNEQAS (Sheffield, UK). The laboratory results were provided directly to the Robertson Centre for Biostatistics, University of Glasgow.

## Cardio-renal MRI

Cardiovascular MRI is the reference diagnostic method for myocardial injury. Cardio-renal MRI was undertaken at a single reference site, the Imaging Centre of Excellence (ICE), Queen Elizabeth University Hospital, University of Glasgow. All patients were imaged on the same research-dedicated MRI and CT scanners rather than on different local hospital clinical service scanners. This approach was intended to minimise measurement variation that might arise during imaging acquisition and analysis.

The patients were scanned using a research-dedicated clinical 3.0 Tesla (3T) MRI scanner (MAGNETOM Prisma, Siemens Healthineers, Erlangen, Germany) with two 18-channel surface coils placed anteriorly and a 32-channel spine coil placed posteriorly in the convalescent phase, 28 – 60 days after discharge. The rationale for undertaking the multi-parametric cardiovascular MRI at this time point was to assess for persisting evidence of cardio-renal injury in the convalescent phase.

## Cardiovascular MRI

Balanced steady-state free precession sequences were used to acquire ventricular cine imaging in three long axis planes, followed by a short axis stack from the apex to the atrioventricular ring, each with 30 phases. Images were obtained using retrospective electrocardiogram-gating at end-expiration. Typical scan parameters were: field of view (FOV) 340 × 286 mm, slice thickness 7 mm with 3 mm gap in short axis stack, repetition time (TR) – 41.4 ms, echo time (TE) 1.51 ms, flip angle 50°, voxel size 1.33×1.33 x 7 mm.

Three left ventricular short axis (basal, mid and apical) and one orthogonal long axis longitudinal relaxation time (T1, spin-lattice relaxation time constant in milliseconds) motion-corrected, optimised, modified Look-Locker inversion recovery sequences^7^ were acquired with the following typical parameters: FOV 360 x 306 mm, slice thickness 8.0 mm, voxel size: 1.9 x 1.9 x 8.0 mm, TR 264 ms, TE 1.12 ms, flip angle 35 degrees, minimum T1 100 ms, inversion-time increment 80 ms, bandwidth 1085 Hertz/pixel.

A short axis stack of transverse relaxation time (T2, spin-spin relaxation time constant in milliseconds) maps and orthogonal long axis views were acquired, followed by an automated exponential fit for each pixel after respiratory motion correction. The imaging used a T2-prepared single shot b-steady-state free precession (SSFP) readout with T2 preparation times (TE) = 0, 25, and 55 ms with a recovery period of 3 heartbeats between measurements. Typical protocol parameters for T2 mapping were: FOV 360 x 270 mm, slice thickness 8 mm, matrix 192 x116, spatial resolution 1.9 x 1.9 mm, TR 207.39 ms, TE 1.32 ms, flip angle 12 degrees, bandwidth 1184 Hz/pixel.

Late gadolinium enhancement images, including three long axis acquisitions and a short axis stack, were acquired 10-15 minutes after intravenous injection of 0.15 mmol/kg of gadolinium diethyltriaminepenta-acetic acid (Gd-DTPA, Magnevist, Bayer Healthcare) using segmented phase-sensitive inversion recovery turbo fast low-angle shot. Typical imaging parameters were: matrix = 192 x 111, flip angle = 14°, TE =1.05 ms, bandwidth =1085 Hz/pixel, echo spacing = 2.1 ms and trigger pulse = 1 ms. The voxel size was 1.9 x 1.9 x 7 mm^3^. Inversion times were individually adjusted to optimise nulling of visually normal myocardium (typical values, 250 to 350 ms).

Three left ventricular short axis (basal, mid and apical) and orthogonal long axis T1 motion-corrected, optimised, modified Look-Locker inversion recovery sequences were acquired 15 minutes after contrast administration with the following typical parameters: FOV 360 x 306 mm, slice thickness 8.0 mm, voxel size: 1.9 x 1.9 x 8.0 mm, TR 341 ms, TE 1.01 ms, flip angle 35 degrees, minimum T1 100 ms, inversion-time (TI) increment 80ms, bandwidth 1085Hertz/pixel.

## Cardiovascular MRI analysis

The cardiovascular MRI scans were reviewed and reported by an accredited radiologist. A single image analyst (K.M. with >8 years of image analyses experience) analysed all data, which were subsequently reviewed by C.B. (with >15 years of image analyses experience).

## Reference ranges

Contemporary, local reference ranges were derived using the 3T MRI scanner (MAGNETOM Prisma, Siemens Healthineers, Erlangen, Germany) by A.M. and K.M. as part of standard quality assurance in the University of Glasgow Clinical Imaging Research Facility. These scans were acquired during the same period as the current study and analysed using dedicated software (cvi42 software for Cardiovascular MRI, version 5.10, Circle Cardiovascular, Canada) to derive mean, upper, and lower reference ranges. This software package was also used for the cardiovascular MRI analyses of the study participants.

## Ventricular function

The imaging analyses were performed utilising dedicated cardiovascular MRI software (cvi42 software (version 5.10, Circle Cardiovascular, Canada)). Routinely reported measures of left ventricular and right ventricular function were carried out according to contemporary guidelines^8^. Ventricular endocardial and epicardial contours were manually drawn at end-diastole and end-systole, which was deemed the phase with the smallest blood pool cavity. Papillary muscles were excluded from myocardial mass and included in volumes. Global left ventricular strain (circumferential, longitudinal, and radial) and global right ventricular strain (longitudinal) were derived using the software's tissue tracking module to determine peak values for each parameter. Atrial areas were manually drawn on 4-chamber horizontal long axis views at atrial diastole (defined with respect to mitral valve closure).

## Parametric mapping

Motion corrected T1, and T2 scans were analysed using dedicated software (cvi42 software (version 5.10, Circle Cardiovascular, Canada). The individual images were reviewed to ensure that motion correction was successful. Parametric maps were generated, and goodness-of-fit (R^2^) was reviewed. Myocardial segments with artefact that impaired diagnostic quality or measurement accuracy, including pixels/segments with R^2^<0.99, were excluded from the analysis.

Epi- and endocardial borders were manually drawn, and care was taken to include only myocardial tissue with a 10% epi- and endocardial offset applied to avoid partial volume effects. The right ventricular insertion points were used to segment the myocardium as per the American Heart Association's 16-segment left ventricular model^9^. For blood pool, pre- and post-contrast T1 regions of interest were drawn within the left ventricular cavity on the three short axis maps. Care was taken to avoid artefacts and papillary muscles.

Haematocrit values were acquired on the day of the study visit. Additional regions of interest were manually drawn on a representative area of serratus anterior, identified from the T2 stack.

## Late gadolinium enhancement imaging

The archive of late gadolinium enhancement images for each patient was initially qualitatively reviewed for image quality and artefacts. The imaging set included the short axis stack and three or more orthogonal long axis views.

Myocardial late gadolinium patterns were predefined. They included myocarditis, myocardial infarction, non-ischaemic cardiomyopathy, and microvascular thrombosis. The location of the late gadolinium enhancement was defined as sub-endocardial, mid-wall, sup-epicardial, or pericardial. Myocardial hyperenhancement in the basal septum was reviewed in association with the cardiac-gated CT image reconstruction. If compatible with a septal perforator artery, this feature was excluded from the late gadolinium enhancement analyses. Hyperenhancement at right ventricular insertion points may be observed in individuals without cardiac disease. Therefore, this feature was not defined as pathological.

The full width at half maximum (FWHM) technique was used to evaluate myocardial late gadolinium enhancement imaging based on a literature review by K.M. as this method is reported to be highly reproducible^10,11^, and less conducive to 'over-reporting' the extent of late gadolinium enhancement when compared with other methods^11,12^. The FWHM technique is the optimal semi-automated quantification method in risk-stratifying patients with suspected myocarditis, demonstrating the strongest association with major adverse cardiac events^11^. Late gadolinium enhancement was reported according to the pattern (distribution) on a per-segment and per-patient basis. The aetiological categories for the pattern of late gadolinium enhancement included non-ischaemic, ischaemic, mixed, micro-thrombi, other, or none. Late gadolinium enhancement was quantified as the percentage of left ventricular mass.

## Renal MRI protocol

Multi-parametric renal MRI included anatomical imaging and mapping native T1 and T2. The volume (ml) and native T1 (ms) and T2 (ms) in regions of interest obtained within the cortex and medulla of each kidney were recorded, and the averaged values of these parameters for both kidneys were then determined. Corticomedullary differentiation reflects a difference in tissue contrast on T1-weighted imaging due to a shorter T1 relaxation time of the cortex relative to the medulla, this being attributed to differences in water content between the two tissues disease^13,14^. Corticomedullary differentiation, reported here as a ratio of T1 cortex divided by T1 medulla^14^, may diminish in kidney disease^13^.

Transverse volumetric interpolated breath-hold examination (VIBE) images, with and without contrast, were acquired to assess kidney volume. For T1 and T2 sequences, single oblique coronal slices positioned through the centre of both kidneys were acquired with breath-held at expiration. The right kidney was prioritised in patients where both kidneys could not clearly be included.

T1 maps were acquired using a modified look-locker inversion recovery (MOLLI) sequence with a single shot true FISP readout. Images were acquired at eight different inversion times (pattern 5(3)3) with a start TI of 180 ms and a TI increment of 80 ms. Motion correction and fitting of the T1 map were performed using a phase-sensitive inversion recovery reconstruction implemented in the vendor software (VE11C, Myomaps, Siemens). Other imaging parameters were: FOV 360*213 mm, slice thickness 5 mm, matrix 240 *190, spatial resolution 1.5*1.5 mm, TE 1.2 ms, flip angle 35 degrees, bandwidth 1096 Hz/pixel. The initial T1 protocol used a repetition time (TR) of 550ms (producing inversion times (TI) of 130, 210, 680, 760, 1230, 1310, 1780, 2330ms) in error. The preferred T1 mapping sequence had a TR of 1000ms to produce a broader range of TIs (TI: 130, 210, 1130, 1210, 2130, 2210, 3130, 4130ms). Once corrected, all subsequent participants were scanned using TR 550 ms and TR 1000 ms protocols. Where available, TR1000 ms was used preferentially in analysis, but participants with only TR550 images were not excluded.

T2 maps were acquired using a fast low angle shot (FLASH) inversion recovery gradient echo sequence, with TR 389 ms, TE 1.4 ms, preparation pulses 0, 30 55 ms, slice thickness 5mm, FOV 360*213 mm, matrix 240*182, spatial resolution 1.5*1.5 mm.

T1 VIBE images were acquired using FLASH inversion recovery gradient echo sequence with TR 3.1 ms, TE 1.22 ms, SPectral Attenuated Inversion Recovery (SPAIR) fat saturation, slice thickness 1.5 mm, FOV 380*308 mm, matrix 256* 192, spatial resolution 1.5*1.5 mm.

## Renal MRI Analysis

Imaging analysis was performed using a custom ImageJ macro (ImageJ, U. S. National Institutes of Health, MD, USA) by K.J.M., P.H.B. and trained physicist colleagues. A thresholding technique was applied to the renal MRI T1 maps to segment the cortex and medulla, creating two regions of interest for T1 (milliseconds), excluding the renal pelvis. These regions of interest were overlaid onto the renal MRI T2 maps allowing measurement of T2 (milliseconds). Renal corticomedullary differentiation was calculated by dividing values for the cortex by those of the medulla. Total kidney volume (ml) was determined by manually tracing the kidney on multiple slices to assess the area, which the software multiplies by slice thickness to determine volume. Overall, the mean values of the measurements for each kidney were taken to represent the left and right values.

## Computed Tomography

One dedicated clinical research CT scanner was used for chest CT, including pulmonary and coronary angiography, 28-60 days post-discharge. A 320-detector CT scanner (Aquilion ONE, Canon) provided full heart coverage within a single heartbeat. Intravenous metoprolol was used where required to control the heart rate (target 60/min), and sublingual glyceryl trinitrate was given to all patients immediately before the scan acquisition. An initial low radiation dose helical scan of the thorax was acquired for a comprehensive assessment of the lungs. A contrast bolus timing scan was acquired to provide information on cardiopulmonary transit times. Non-contrast and contrast-enhanced angiographic breath-hold ECG-gated volumes were acquired timed for optimum pulmonary and systemic arterial (coronary) opacification.

Coronary CT angiography provided information on the presence and extent of coronary calcification (calcium score) according to the Agatston and Multi-Ethnic Study of Atherosclerosis (MESA) scores^15^. The Agatston score is a semi-automated tool to calculate a score based on the extent of coronary artery calcification detected by an unenhanced, non-contrast, low-dose CT scan. The score is generated using a formula that considers the area and the peak density of calcified lesions. The score sets a threshold of >130 Hounsfield units with an area of three or more pixels to identify calcified coronary atherosclerosis. The calcium score may be categorised into minimal (0–10), mild, moderate (100–400), or severe (400) and can be plotted against age- and gender-specific nomograms to determine the calcium score percentile. The values increase with age and are higher for men than women in all age groups except the extremely elderly. The MESA score provides the estimated probability of non-zero calcium and the 25th, 50th, 75th, and 90th percentiles of the calcium score distribution for a particular age, gender and race. Using the observed calcium score revealed by CT coronary angiography, the percentile for this score can then be estimated. Coronary artery disease and whether any coronary artery disease was obstructive by angiographic criteria, including the CAD-RADS score^16^. Coronary geometries derived from coronary CT angiography can be utilised to perform blood flow simulation and estimate myocardial fractional flow reserve (FFR). The functional significance of coronary artery disease was evaluated using FFR_CT_, (HeartFlow, Redwood City, CA). FFR_CT_ technology involves proprietary software with quantitative image quality analysis, image segmentation, physiological modelling, and artificial intelligence automation. FFR_CT_ is accurate compared with invasive FFR^17^. FFR_CT_ was calculated prospectively, independent of the research team and blind to the other clinical data.

Late contrast enhancement ECG-gated CT was acquired to assess for delayed enhancement (scar)^18^ and ECV calculation^19^. Pulmonary vascular imaging assessed pulmonary vascular thrombosis (embolism), including CT obstruction score, cardiopulmonary transit times and measures of raised pulmonary artery & right heart pressures (pulmonary artery, caval and azygous dimensions plus hepatic inferior vena cava reflux). CT was used to characterise pulmonary features associated with COVID infection, e.g. ground glass opacity and/or consolidation, and pre-existing lung damage e.g., emphysema. The CT and MRI findings were correlated with the clinical data. Cardiac and extra-cardiac findings were reported and managed according to local standards of care. Patients with severe renal dysfunction thought to be at risk of acute kidney injury as determined by local Radiology clinical protocols underwent non-contrast CT.

## Blinding

The patients and the outcome assessors were blinded. Outcome assessments, including laboratory, MRI and CT analyses and endpoint adjudication, were undertaken by blinded researchers. The patients completed the questionnaires before undergoing the scans and were unaware of the test results.

## Gluteal biopsies

Biopsies were performed on prone patients using 2% lidocaine local anaesthetic in a sterile surgical field. A 4-6cm^2^ sample of subcutaneous fat with 0.4cm^2^ skin was excised and submerged in physiological saline solution. Intact small arteries (< 500μm) were dissected from this subcutaneous fat. These arteries were used for histopathology, functional (wire myography), and molecular studies and vascular smooth muscle cells (VSMCs) were isolated for primary cell culture, as previously described and summarised below^20^. Identical protocols were used for laboratory studies in tissues obtained from post-COVID-19 patients and controls. Pharmacological assessment of peripheral vascular function was performed at least three months after COVID-19 illness, beyond the initial infection phase and inflammatory response.

## Human vascular functional studies

Small arteries were dissected from gluteal fat and cut into 2 mm ring segments. Arterial segments were mounted on isometric wire myographs (Danish Myo Technology, Denmark) filled with 5ml of physiological saline solution [(in mmol/L: 130 NaCl, 14.9 NaHCO_3_, 4.7 KCl, 1.18KH_2_PO_4_, 1.17 MgSO_4•_7H_2_O, 5.5 glucose, 1.56 CaCl_2•_2H_2_O, and 0.026 EDTA] and continuously gassed with a mixture of 95% O_2_ and 5% CO_2_ while being maintained at a constant temperature of 37±0.5^º^C. Following 30 minutes of equilibration, the contractile responses of arterial segments were assessed by adding KCl (62.5mmol/L). Blood vessels with no responses were retained for cell culture or molecular studies. The integrity of the endothelium was verified by relaxation induced by acetylcholine (ACh, 10^-6^ mol/L) in arteries pre-contracted with U46619 (thromboxane-A2 analogue, 10^-7^ mol/L). Cumulative concentration-response curves assessed endothelium-dependent relaxation to ACh (10^-9^-3x10^-5^ mol/L) in human vessels. Concentration-response curves assessed endothelium-independent vasorelaxation to sodium nitroprusside (SNP; 10^-10^- 10^-5^ mol/L) in human vessels. Concentration-response curves to U46619 (10^-10^ – 10^-6^ mol/L) and endothelin-1 (ET-1; 10^-12^ – 10^-7^ mol/L) were performed to evaluate vasoconstriction in human arteries. Vascular functional responses were also assessed in the absence and presence of a rho-kinase inhibitor, fasudil (10^-6^ mol/L, 30 minutes). The vascular sensitivity (pEC_50_) and maximum responses (E_max_) to each agonist were determined using Labchart^®^ ADInstruments.

## Spatial Transcriptomics

Spatial transcriptomics (Nanostring GeoMx Digital Spatial Profiler (DSP)) was used to assess the distribution of gene expression in small artery sections. Whole transcriptome profiling of the vascular wall was performed using formalin-fixed paraffin-embedded (FFPE) tissue sections (5 μm) in triplicate for one COVID-19 patient and one age, sex, and cardiovascular risk factor matched control. Whole regions of interest (ROIs) were collected; no segmentation was performed. Bioinformatics analysis was performed on the native GeoMx DSP Data Analysis Suite before using additional custom R pipeline analysis to aid visualisation. Whole ROIs were collected; no segmentation was performed.

For GeoMx DSP slide preparation and sample collections, the slide preparation user manual (MAN-10087-04) was followed. In brief, tissue slides were baked in a drying oven at 60 °C for one hour and then loaded to Leica Biosystems BOND RX FFPE for deparaffinisation and rehydration. Following antigen retrieval, tissues were treated with 0.1ug/ml proteinase K solution to expose ribonucleic acid (RNA) targets, followed by fixation with 10% neutral buffered formalin (NBF). After all tissue pre-treatments, tissue slides were incubated with an RNA probe mix (Human Whole Transcriptome Atlas; a pool of in-situ hybridisation probes with UV photocleavable oligonucleotide barcodes)

Tissues were washed in a 1:1 solution containing 4X saline-sodium citrate (SSC) and formamide before being stained with fluorophore-conjugated primary antibodies: PanCK-532 (NanoString Technologies, 1:40), CD45-594 (NanoString Technologies, 1:40) and CD31-647 (Abcam, 1:200). SYTO13 (NanoString Technologies, 1:10) was employed for nuclear visualisation. We followed the user manual for GeoMx DSP sample collections (MAN-10088-03). In brief, tissue slides were loaded on the GeoMx DSP instrument and then scanned according to manufacturer recommendations to visualise the tissue regions, all measuring approximately 650um in diameter.

Each GeoMx DSP sample (n=6) plus non-template controls (NTCs) were uniquely indexed using pre-prepared primers ('Seq Codes', Nanostring), which are compatible with the dual-indexing system from Illumina. Thermocycler conditions were 37 °C (30 min), 50 °C (10 min), 95 °C (3 min), 18 cycles of 95 °C (15 sec), 65 °C (60 sec), 68 °C (30 sec), and final extension of 68 °C (5 min). Following library preparation, PCR reactions were pooled (according to each seq code plate) and purified using AMPure XP beads (Beckman Coulter) as per the manufacturer's instructions. Libraries were paired-end sequenced (2 × 27bp) on a NextSeq2000. FASTQ files were thereby generated for each DSP collection. Raw data were demultiplexed and converted to digital count conversion (DCC) files using Nanostring's GeoMx DnD pipeline (v.1).

## Data processing and normalisation

DCC files were uploaded onto the GeoMx Analysis suite for QC and upper quartile (Q3) normalisation. A minimum of 10,000 reads were required for each non-NTC sample. Probes were checked for outlier status by implementing a global Grubb's outlier test with alpha set to 0.01. Data were then normalised to the geometric mean of the 75th percentile across all ROIs to give the Q3 normalisation factors for each ROI. The distribution of these Q3 normalisation factors was then checked for outliers defined as any ROI greater than two s.d. from the mean log^2^-transformed Q3 normalisation factor. All ROIs were included in the downstream analysis; according to the quality control criterion as described by Nanostring.

## Histopathology and immunohistochemistry

Fresh, vascular samples were formalin-fixed and impregnated with paraffin before staining. Analyses were undertaken using a de-identified dataset blind to COVID-19 status and the results of the other vascular investigations performed.

### Histopathology

Masson’s Trichrome staining was used to selectively stain connective tissue including collagen, from cells. Wiegert’s haematoxylin was used to stain cell nuclei. Plasma stain was then applied, followed by phosphomolybdic acid and aniline blue. Picrosirius red staining was also performed on additional sections, with celestine blue staining, Wiegert’s haematoxylin, acid alcohol differentiation and Sirius Red stain. Slides were scanned in high resolution for digital analysis in Image J (Fiji v1.53f51) at 40x magnification. Colour deconvolution for Masson Trichrome and picrosirius red was performed with threshold adjustment to assess the percentage of stain by colour for each vascular sample (**Figure S1**). The proportion of aniline blue or picrosirius red stained tissue from all tissue within the region of interest was then calculated and compared between post-COVID-19 and control samples.

### Antigen retrieval and immunohistochemistry

Heat-induced epitope retrieval was performed for antigen retrieval, with sections treated at full pressure with Access Retrieval Unit (Menarini) in a sodium citrate buffer for anti-myosin light chain (phosphor S20) antibody (abcam 2480). Sections were then washed in Tris Tween buffer.

Hydrogen peroxide (3%) treatment was then applied in phosphate buffered saline to quench peroxidase activity, followed by two further washes with TRIS Tween buffer. Sections were then incubated at room temperature for 30 minutes with the primary antibody anti-myosin chain (abcam) at 1:400 concentration. A further wash with TRIS Tween buffer was then performed.

To detect primary antibodies, the sections were then incubated with EnVision+ System HRP Labelled Polymer Anti-Rabbit Secondary Antibody (Dako) for 30 minutes at room temperature. A further washing with TRIS Tween buffer was performed followed by two, five-minute incubations with 3,3’=diaminobenzidine (DAB) substrate-chromogen (EnVision+ System, Dako). Sections were then rinsed twice for five minutes in distilled water prior to being counterstained using Gill’s haematoxylin and mounted using DPX mounting media (Cellpath). Slides were scanned in high resolution for digital analysis in Image J (Fiji v1.53f51) at 40x magnification. A colour deconvolution to calculate the total area and proportion of tissue within the slide was performed. The slide image was then reset, and converted to 8-bit for threshold analysis of positively stained tissue area. The proportion of positively stained tissue to total tissue was then calculated and compared between COVID-19 and control group.

**Transcriptomic Data Analysis**

Bioinformatics analysis was performed on the native GeoMx DSP Data Analysis Suite before using additional custom R pipeline analysis to aid visualisation. Normalised counts were downloaded into RStudio (v2022.02.2+485) using R build version 4.2.1. Differential Gene Expression (DGE) was performed using the edgeR package with Fisher’s Exact test used to determine statistical significance^21^. Volcano plots were generated using the EnhancedVolcano package^22^. An unsupervised analysis of intrinsic patterns of gene expression was performed using the weighted gene co-expression network analysis (WGCNA) package^23^. Gene set module enrichment was performed using the ClusterProfiler package^24^ using Reactome and Gene Ontology as reference. Immune cell counts were extracted from transcriptome using the SpatialDecon tool^25^. Normalised counts and source code are available on request.

## Statistical analysis

Cumulative concentration-response curves (CCRCs) were fitted using a four-parameter, non-linear regression curve fitting in GraphPad Prism 8.0 (GraphPad Inc, USA). Maximum efficacy (Emax) for vasoconstrictors is expressed as a % of the mean response of the contraction to 62.5mM KCl. For relaxation data, the maximum response (Emax) to ACh and SNP are expressed as % relaxation after pre-constriction with U46619 (0.1μM). The sensitivity of the arteries to each compound was expressed as the pEC50 (constrictors) or pIC50 (inhibitors) derived from the CCRC using GraphPad Prism 8.0. The pEC50 value represents the minus log concentration required to produce 50% of the maximum response.

Similarly, pIC50 is the -log of concentration required to inhibit 50% of the maximum response. Higher numbers indicate more potency (less concentration needed to achieve the median response). The pEC50 values were calculated by computer interpolation from individual CCRCs.

Data were expressed as mean±SEM unless otherwise stated. Statistical comparisons of continuous parameters between groups were performed using two-tailed student's t-test, 1- way and 2-way ANOVA, followed by Bonferroni post hoc tests as appropriate. Fisher's exact tests compare categorical variables within demographics and clinical data. P <0.05 was considered statistically significant. Repeated measures ANOVA was used to compare groups within vascular reactivity studies.

## Sample size calculation

The primary endpoint of this study was the difference in maximum contraction (Emax) induced by U46619 between the two groups. Using preliminary vascular reactivity data from gluteal biopsies from microvascular angina vs control patients^26^, we assumed a meaningful difference between the mean values in the experimental and control groups as 21.1 and standard deviation of 15. Using significance level of 0.05 and level of power as 80%, a minimum sample size of eight per group was estimated using G*Power 3.1 (University of Melbourne, Parkville, Victoria, Australia). This calculation was based on the Mann–Whitney U test reflecting a small sample size and the likelihood of non-parametric distribution.

## Clinical vignettes for patients included in the spatial transcriptomics: (A) post-COVID-19, and (B) control without a history or laboratory evidence of COVID-19.

**Patient with convalescent COVID-19 (01-185)**

A 50-year-old female healthcare worker was hospitalised with increasing shortness of breath, cough and pyrexia. There was no history of chest pain. The medical history included hypothyroidism. A PCR test was positive for COVID-19. Chest x-ray at admission revealed Bibasal consolidation with associated effusion. Covid pneumonitis treatment included oxygen, remdesivir and dexamethasone. The patient was initially transferred to a medical high dependency unit for non-invasive ventilation. Due to respiratory distress, hypoxia and delirium, the patient was intubated and mechanically ventilated. A computed tomography (CT) pulmonary angiogram was performed because of tachycardia and rising oxygen requirement, revealing COVID-19 pneumonitis with no evidence of pulmonary thromboembolism. The patient developed melaena during a two-week intensive care admission whilst anticoagulated necessitating transfusion and intravenous proton pump inhibitor infusions. Peak C-reactive protein during hospitalisation was 280 mg/L, and peak high sensitivity troponin-I was 6 ng/L. The research CT scan revealed resolving changes of the lung parenchyma. Coronary CT angiography with CT fractional flow reserve excluded obstructive coronary disease. Cardio-renal magnetic resonance imaging revealed increased myocardial T2 and T1 relaxation times, meeting Lake Louise criteria for myocardial inflammation. There was no late gadolinium enhancement within the myocardium. Left ventricular ejection fraction was preserved, and stress-perfusion revealed preserved global myocardial blood flow (2.52 mL/min/g) with borderline myocardial perfusion reserve (2.0). At the research imaging visit, performed 69 days following COVID-19 diagnosis, and 53 days following discharge from hospital, the physical and psychological function scores using patient-reported outcome measures were impaired including the Duke Activity Status Index calculated at 27 (VO­_2_ peak 21 ml/kg). The gluteal biopsy was undertaken 153 days after the diagnosis of acute COVID-19.

**Control patient (01-232)**

A 48-year-old female health care worker presented to the cardiology clinic with six months of chest pain. History of presenting complaint described an exertional component on mild inclines and mild ankle oedema. A referral was placed for clinical CT coronary angiography, and the patient was highlighted for approach by the study team. Medical history included previous smoking status and increased body mass index (36 kg/m^2^), and a family history of premature ischaemic heart disease. Baseline clinical investigations, including chest x-ray and electrocardiogram, were normal, and the patient's blood pressure was 134/63 mmHg. High sensitivity troponin I was <4 ng/L, and C-reactive protein was 5mg/L. Research imaging CT coronary angiogram demonstrated no coronary disease. T2 and T1 relaxation times were within reference ranges, left ventricular ejection fraction was preserved, and stress-perfusion revealed global stress myocardial blood flow of 2.2 mL/min/g (abnormal <1.94 mL/min/g) with normal myocardial perfusion reserve (3.0). The physical and psychological function scores using patient-reported outcome measures were normal, including the Duke Activity Status Index of 58.2 (VO_2_ peak 34.6 ml/kg).

# Supplement References

1. Morrow AJ, Sykes R, McIntosh A, Kamdar A, Bagot C, Bayes HK, *et al*. A multisystem, cardio-renal investigation of post-COVID-19 illness. *Nat Med* 2022.

2. Mangion K, Morrow A, Bagot C, Bayes H, Blyth KG, Church C, *et al*. The Chief Scientist Office Cardiovascular and Pulmonary Imaging in SARS Coronavirus disease-19 (CISCO-19) study. *Cardiovasc Res* 2020;**116**:2185–2196.

3. Coronavirus (COVID-19) clinical radiology resources | The Royal College of Radiologistshttps://www.rcr.ac.uk/college/coronavirus-covid-19-what-rcr-doing/coronavirus-covid-19-clinical-radiology-resources (25 August 2021)

4. Chung M, Bernheim A, Mei X, Zhang N, Huang M, Zeng X, *et al*. CT Imaging Features of 2019 Novel Coronavirus (2019-nCoV). *Radiology* 2020;**295**:202–207.

5. Levey AS, Stevens LA, Schmid CH, Zhang Y (Lucy), Castro AF, Feldman HI, *et al*. A New Equation to Estimate Glomerular Filtration Rate. *Ann Intern Med* 2009;**150**:604–612.

6. Broch K, Eek C, Wergeland R, Ueland T, Skårdal R, Aukrust P, *et al*. NT-proBNP predicts myocardial recovery after non-ST-elevation acute coronary syndrome. *Scand Cardiovasc J SCJ* 2012;**46**:65–71.

7. Messroghli DR, Radjenovic A, Kozerke S, Higgins DM, Sivananthan MU, Ridgway JP. Modified Look-Locker inversion recovery (MOLLI) for high-resolution T1 mapping of the heart. *Magn Reson Med* 2004;**52**:141–146.

8. Petersen SE, Aung N, Sanghvi MM, Zemrak F, Fung K, Paiva JM, *et al*. Reference ranges for cardiac structure and function using cardiovascular magnetic resonance (CMR) in Caucasians from the UK Biobank population cohort. *J Cardiovasc Magn Reson* 2017;**19**:18.

9. Cerqueira MD, Weissman NJ, Dilsizian V, Jacobs AK, Kaul S, Laskey WK, *et al*. Standardized myocardial segmentation and nomenclature for tomographic imaging of the heart. A statement for healthcare professionals from the Cardiac Imaging Committee of the Council on Clinical Cardiology of the American Heart Association. *Circulation* 2002;**105**:539–542.

10. Flett AS, Hasleton J, Cook C, Hausenloy D, Quarta G, Ariti C, *et al*. Evaluation of Techniques for the Quantification of Myocardial Scar of Differing Etiology Using Cardiac Magnetic Resonance. *JACC Cardiovasc Imaging* 2011;**4**:150–156.

11. Gräni C, Eichhorn C, Bière L, Kaneko K, Murthy VL, Agarwal V, *et al*. Comparison of myocardial fibrosis quantification methods by cardiovascular magnetic resonance imaging for risk stratification of patients with suspected myocarditis. *J Cardiovasc Magn Reson* 2019;**21**:14.

12. Khan JN, Nazir SA, Horsfield MA, Singh A, Kanagala P, Greenwood JP, *et al*. Comparison of semi-automated methods to quantify infarct size and area at risk by cardiovascular magnetic resonance imaging at 1.5T and 3.0T field strengths. *BMC Res Notes* 2015;**8**.

13. Wolf M, Boer A de, Sharma K, Boor P, Leiner T, Sunder-Plassmann G, *et al*. Magnetic resonance imaging T1- and T2-mapping to assess renal structure and function: a systematic review and statement paper. *Nephrol Dial Transplant* 2018;**33**:ii41–ii50.

14. Dekkers IA, Boer A de, Sharma K, Cox EF, Lamb HJ, Buckley DL, *et al*. Consensus-based technical recommendations for clinical translation of renal T1 and T2 mapping MRI. *Magma N Y N* 2020;**33**:163–176.

15. McClelland RL, Chung H, Detrano R, Post W, Kronmal RA. Distribution of coronary artery calcium by race, gender, and age: results from the Multi-Ethnic Study of Atherosclerosis (MESA). *Circulation* 2006;**113**:30–37.

16. Cury RC, Abbara S, Achenbach S, Agatston A, Berman DS, Budoff MJ, *et al*. CAD-RADS^TM^: Coronary Artery Disease - Reporting and Data System: An Expert Consensus Document of the Society of Cardiovascular Computed Tomography (SCCT), the American College of Radiology (ACR) and the North American Society for Cardiovascular Imaging (NASCI). Endorsed by the American College of Cardiology. *J Am Coll Radiol JACR* 2016;**13**:1458-1466.e9.

17. Nørgaard BL, Leipsic J, Gaur S, Seneviratne S, Ko BS, Ito H, *et al*. Diagnostic performance of noninvasive fractional flow reserve derived from coronary computed tomography angiography in suspected coronary artery disease: the NXT trial (Analysis of Coronary Blood Flow Using CT Angiography: Next Steps). *J Am Coll Cardiol* 2014;**63**:1145–1155.

18. Ohta Y, Kitao S, Yunaga H, Fujii S, Mukai N, Yamamoto K, Ogawa T. Myocardial Delayed Enhancement CT for the Evaluation of Heart Failure: Comparison to MRI. *Radiology* 2018;**288**:682–691.

19. Scully PR, Bastarrika G, Moon JC, Treibel TA. Myocardial Extracellular Volume Quantification by Cardiovascular Magnetic Resonance and Computed Tomography. *Curr Cardiol Rep* 2018;**20**:15.

20. Alves-Lopes R, Neves KB, Anagnostopoulou A, Rios FJ, Lacchini S, Montezano AC, Touyz RM. Crosstalk Between Vascular Redox and Calcium Signaling in Hypertension Involves TRPM2 (Transient Receptor Potential Melastatin 2) Cation Channel. *Hypertension* 2020;**75**:139–149.

21. Robinson MD, McCarthy DJ, Smyth GK. edgeR: a Bioconductor package for differential expression analysis of digital gene expression data. *Bioinforma Oxf Engl* 2010;**26**:139–140.

22. Blighe K. EnhancedVolcano: publication-ready volcano plots with enhanced colouring and labeling

23. Langfelder P, Horvath S. WGCNA: an R package for weighted correlation network analysis. *BMC Bioinformatics* 2008;**9**:559.

24. Wu T, Hu E, Xu S, Chen M, Guo P, Dai Z, *et al*. clusterProfiler 4.0: A universal enrichment tool for interpreting omics data. *Innov Camb Mass* 2021;**2**:100141.

25. Griswold M, Danaher P. SpatialDecon: Deconvolution of mixed cells from spatial and/or bulk gene expression data

26. Ford TJ, Rocchiccioli P, Good R, McEntegart M, Eteiba H, Watkins S, *et al*. Systemic microvascular dysfunction in microvascular and vasospastic angina. *Eur Heart J* 2018;**39**:4086–4097.

Table S1. **Clinical outcomes.**

|  |  | All |  | Control | COVID-19 | p-value |
| --- | --- | --- | --- | --- | --- | --- |
|  | | N=37 |  | N=10 | N=27 |  |
| Death or Hospitalisation (Any Cause) | | 6 (16%) |  | 0 (0%) | 6 (22%) | 0.117 |
| Death (Any Cause) | | 0 (0%) |  | 0 (0%) | 0 (0%) | - |
| Hospitalisation (Any Cause) | | 6 (16%) |  | 0 (0%) | 6 (22%) | 0.117 |
| Cardiovascular Hospitalisation | | 3 (8%) |  | 0 (0%) | 3 (11%) | 0.283 |
| Respiratory Hospitalisation | | 0 (0%) |  | 0 (0%) | 0 (0%) | - |
| *Cardiovascular Outcomes* | | | | | | |
| Myocardial infarction | | 0 (0%) |  | 0 (0%) | 0 (0%) | - |
| Percutaneous Coronary Intervention | | 1 (3%) |  | 0 (0%) | 1 (4%) | 0.543 |
| Deep vein thrombosis | | 1 (3%) |  | 0 (0%) | 1 (4%) | 0.543 |
| New atrial fibrillation | | 1 (3%) |  | 0 (0%) | 1 (4%) | 0.543 |
| Ventricular tachycardia or fibrillation | | 0 (0%) |  | 0 (0%) | 0 (0%) | - |
| *Respiratory Outcomes* | | | | | | |
| Pulmonary fibrosis | | 4 (11%) |  | 0 (0%) | 4 (15%) | 0.410 |
| Pulmonary embolism | | 1 (3%) |  | 0 (0%) | 1 (4%) | 0.543 |
| Long-term oxygen therapy | | 1 (3%) |  | 0 (0%) | 1 (4%) | 0.543 |
| *Secondary Care (Outpatients)* | | | | | | |
| Any Outpatient Referral | | 24 (65%) |  | 2 (20%) | 22 (81%) | 0.069 |
| Cardiology | | 2 (5%) |  | 0 (0%) | 2 (7%) | 0.385 |
| Respiratory | | 13 (35%) |  | 0 (0%) | 13 (48%) | **0.024** |
| Physiotherapy | | 7 (19%) |  | 0 (0%) | 7 (26%) | 0.117 |
| Control patients were followed up for a median (interquartile range) of 210 (203, 204) days and COVID-19 patients for 396 (356, 422) days. Clinical outcomes were summarised as numbers and percentages with at least one event and compared between groups using the log-rank test of time to the first event. | | | | | | |

## Figure legend

Figure S1. Histopathology of small, peripheral arteries from a COVID-19 patient demonstrating colour deconvolution technique. ImageJ colour deconvolution windows adjusted for Masson’s Trichrome stain and adjusted for colour thresholds. Colour deconvolution thresholds: Colour 1: red – 0.799511, green – 0.591352, blue – 0.105287; Colour 2: red – 0.099972, green – 0.727286, blue - 0.668033; Colour 3: red – 0.592274, green – 0.326442, blue 0.736646.

Figures S2A & S2B. Vascular reactivity of gluteal biopsies-isolated small vessels from control and COVID-19 patients with no history of hypertension. Cumulative concentration-response curves to (A) U46619 (Thromboxane A2 analogue) (control n=5; COVID-19 n=15) (p=0.043) and (B) sodium nitroprusside (endothelium-independent vasodilator) (control n=6; COVID-19 n=13) (p=0.043) in small arteries isolated from gluteal biopsies derived from control and COVID-19 patients. Relaxant responses were expressed as percentage of U46619-induced pre-constriction and contraction as percentage of KCl responses. Results are expressed as mean±SEM. * vs. control; # vs. COVID-19.

Figures S3A & S3B. Vasomotor function of small arteries isolated from gluteal biopsies obtained from control patients. Cumulative concentration-response curves to (A) U46619 (Thromboxane A2 analogue) (p=0.437) and (B) sodium nitroprusside (endothelium-independent vasodilator) in the presence of fasudil (Rho-kinase inhibitor; 1µmol/L; 30 min) (p=0.224) in small arteries isolated derived from control patients. Relaxant responses were expressed as % of U46619-induced pre-constriction and contraction as % of KCl responses. Results are expressed as mean±SEM (n=3-8).

**Figures S4A & S4B.** Increased collagen deposition was revealed by Masson's Trichrome (MT) and picrosirius red (PSR) staining in small, peripheral arteries isolated from COVID-19 patients (red; MT - n = 26, PSR - n = 26) and age, sex and cardiovascular risk factor matched controls (blue, MT - n = 10, PSR - n = 9). A Box and whiskers plot demonstrating percentage area of aniline blue (**Figure** **S4A**) and PSR (**Figure** **S4B**) staining of collagen between COVID-19 patients (red) and controls (blue) is provided in addition to an example of a vessel from a patient following COVID-19 with an age, sex and cardiovascular risk factor matched control patient vessel. Magnification x40. The mean tissue proportions of aniline blue stain uptake in COVID-19 and control samples were 69.7% (95% CI: 67.8, 71.7) and 64.9% (95% CI: 59.4, 70.3), respectively, equating to a mean difference of 4.9% (95% CI: 0.7, 9.0; p=0.029). The mean tissue proportion of picrosirius red stain uptake in COVID-19 samples was 68.6% (95% CI: 64.4, 72.8) with a mean difference of 8.5% (95% CI: 1.0, 16.0) compared with 60.1% uptake in controls (95% CI: 55.4, 64.8) (p=0.028).

Figure S5. Vascular gene set enrichment analysis. Gene set enrichment analysis undertaken in multiple slices of a small peripheral artery from a patient following COVID-19 compared with age, sex, and cardiovascular risk factor matched control artery.

Figures S6. Spatial deconvolution following whole transcriptome analysis of cell types within a small, peripheral resistance arteries (n=2) obtained from a patient 153 days following COVID-19 (01-185) compared with age, sex, and cardiovascular risk factor matched control (01-232).

# Supplement figures


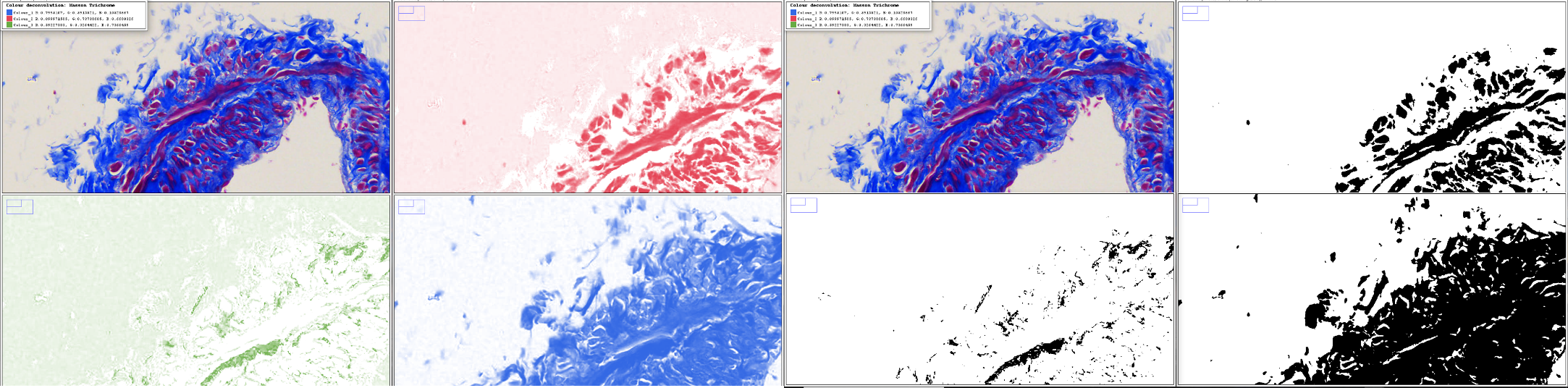
Figure S1.

## Figure S2.


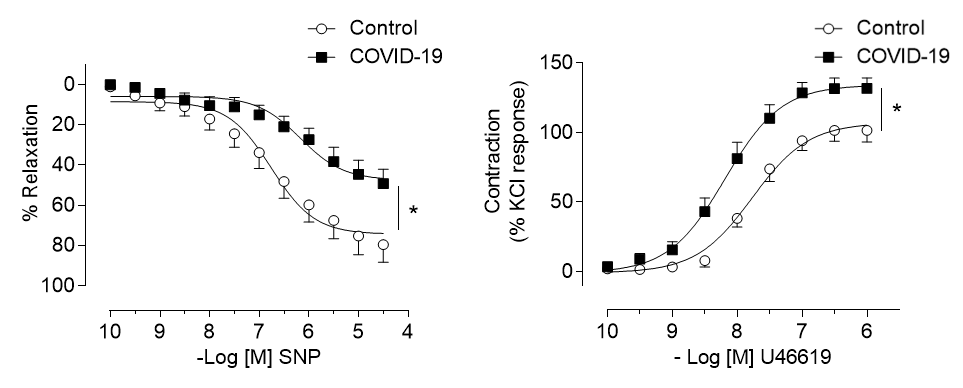


**A**

**B**

Figure S3.

**Figure S4A.**
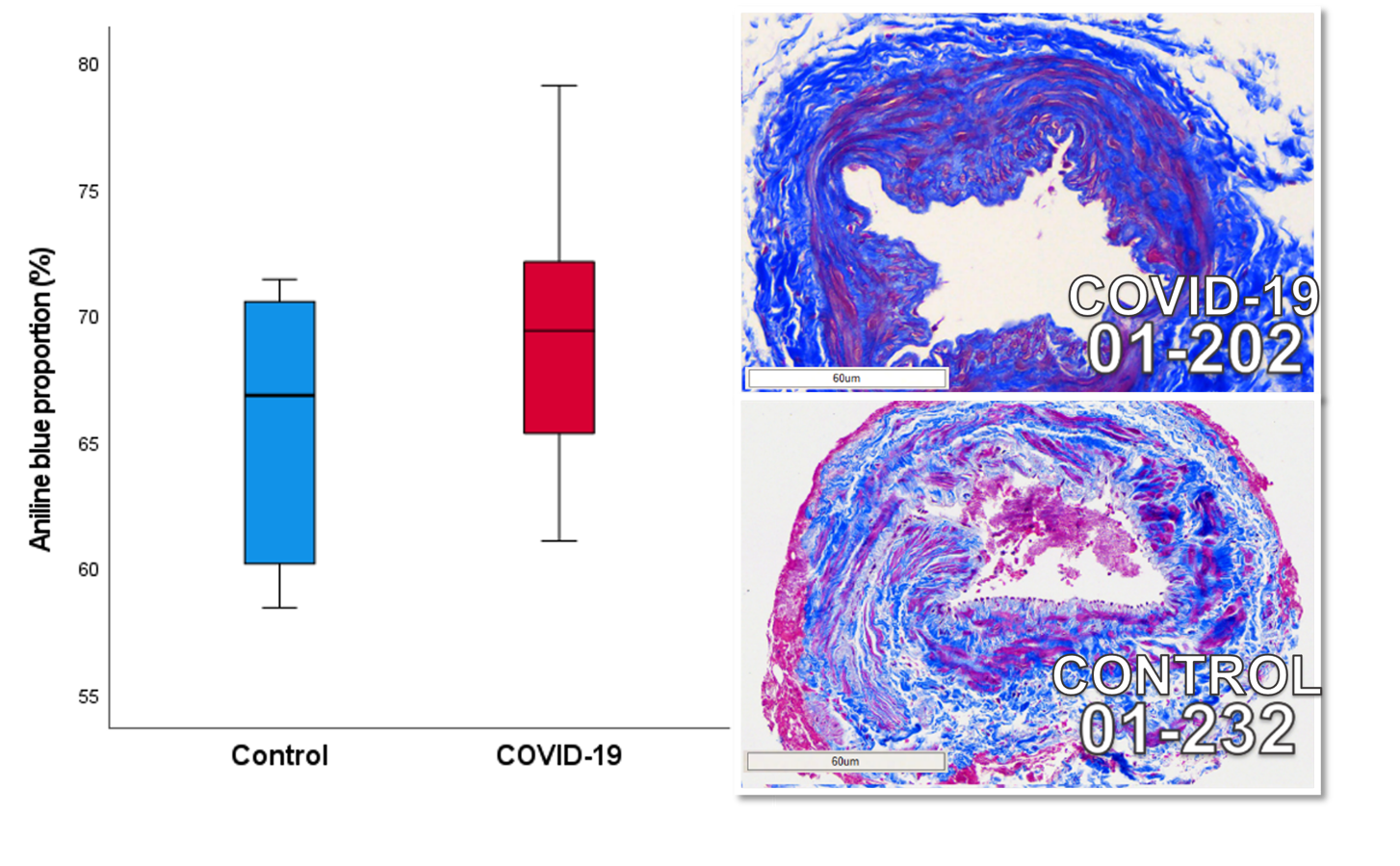


##
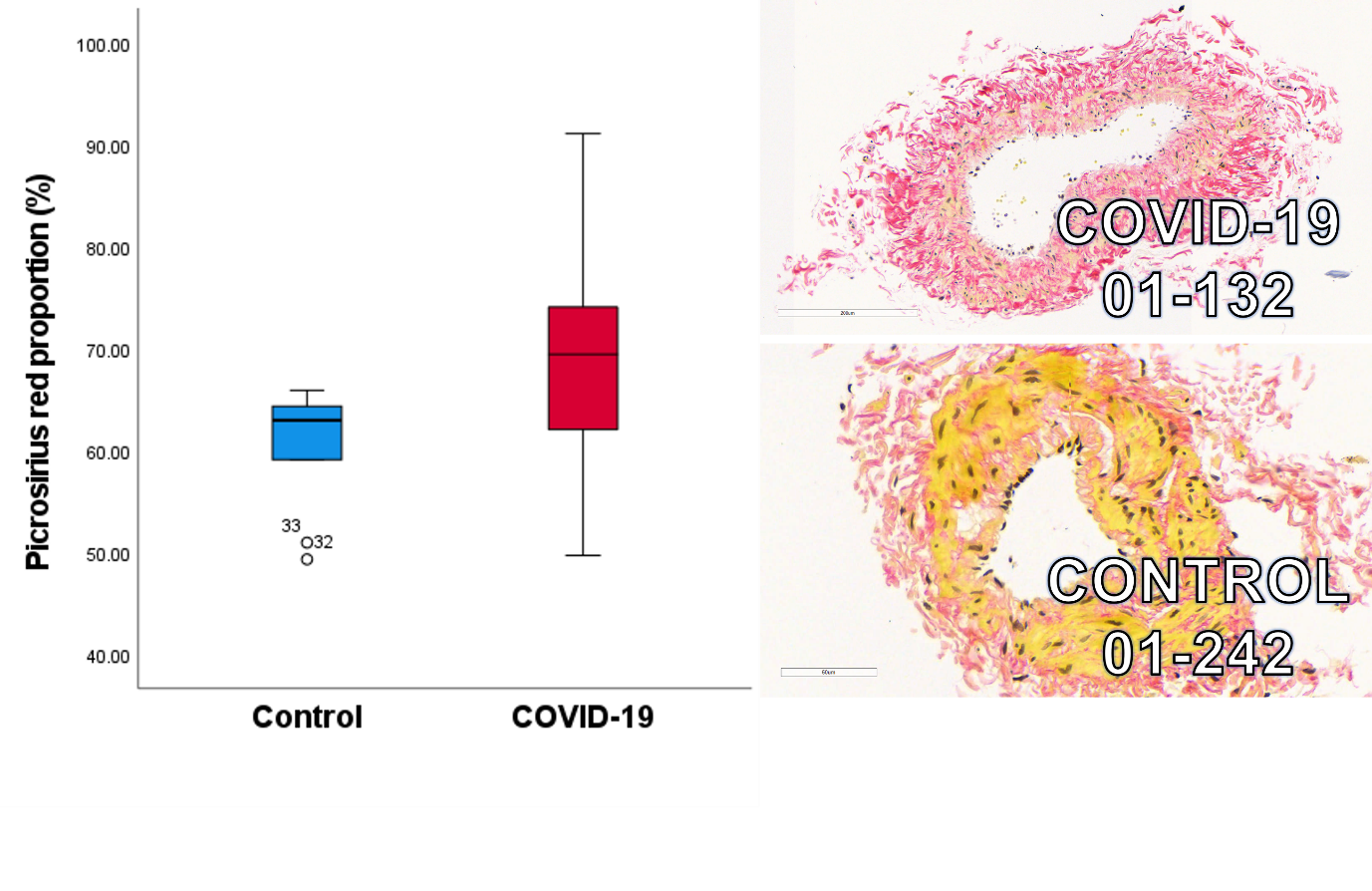
Figure S4B.


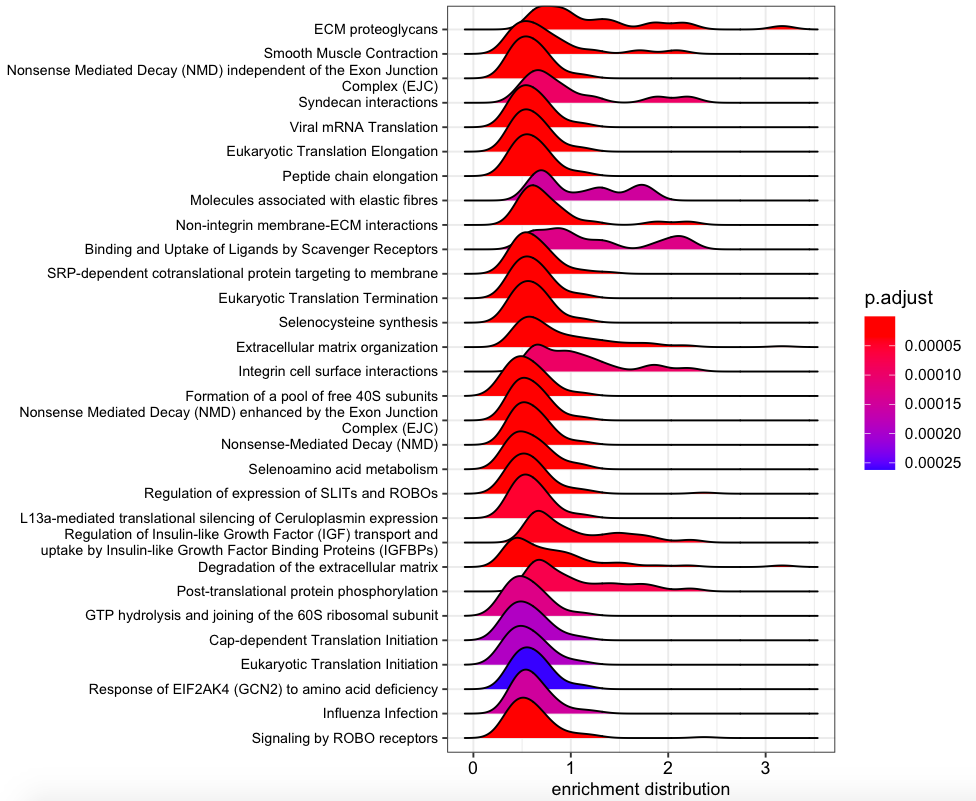

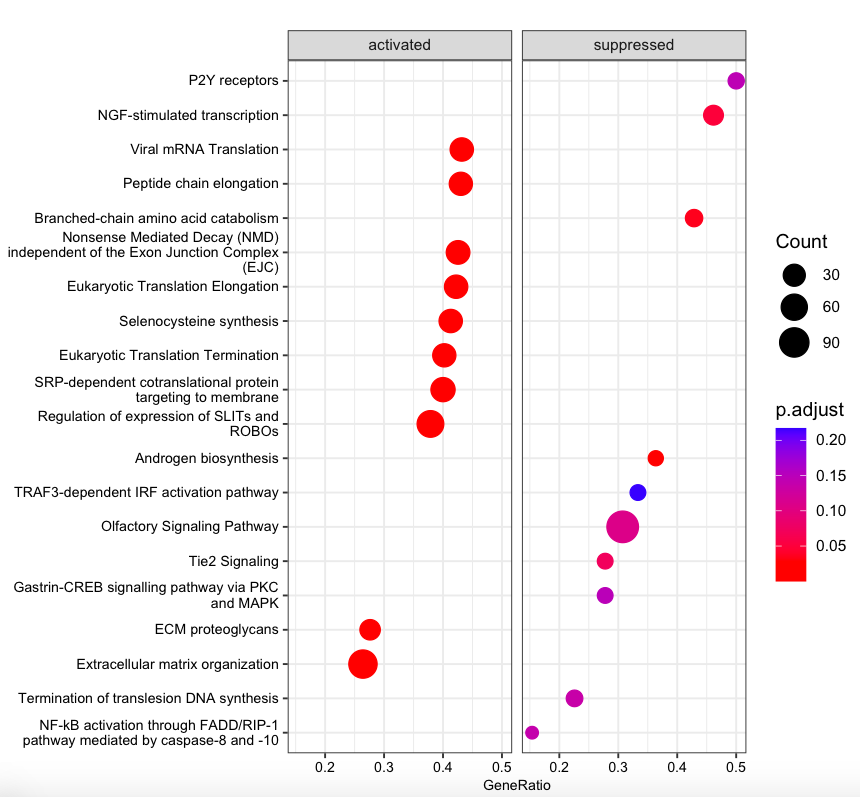
 Figure S5.

Figure S6**.** **
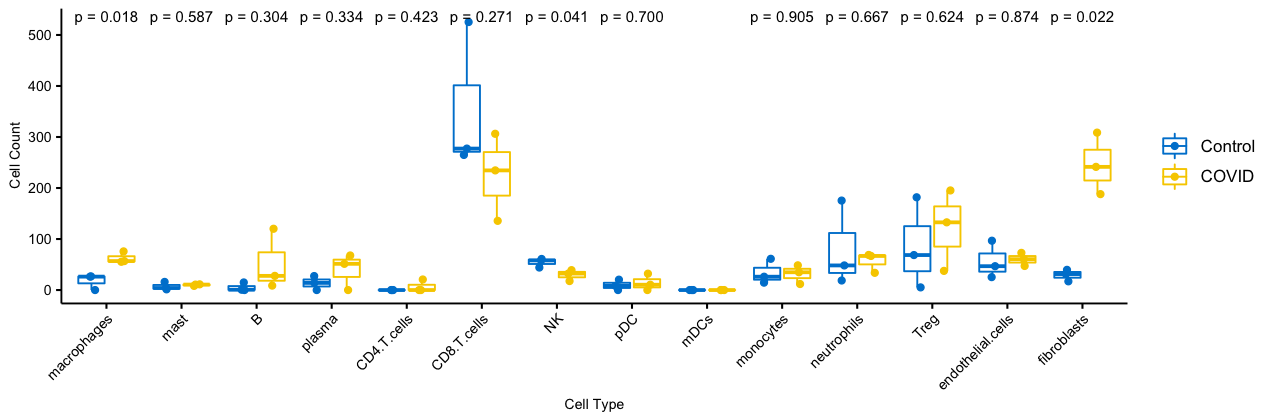
**
